# Supplementary material for: Staggered circular nanoporous graphene converts electromagnetic waves into electricity
Source: Nat Commun. 2023 Apr 8;14:1982. doi: 10.1038/s41467-023-37436-6 (PMC10082851; doi:10.1038/s41467-023-37436-6)
Supplement: Supplementary file 1 — Supplementary Information [file 41467_2023_37436_MOESM1_ESM.pdf]

Table of Contents

Supplementary Notes 3

Supplementary Note 1. Analysis of the low-frequency polarization relaxation of ordered nanoporous graphene 3

Supplementary Note 2. Polarization behavior of graphene with different structures 5

Supplementary Note 3. Effect of pore structure on the polarization intensity of graphene 6

Supplementary Note 4. Calculation of lattice and electron thermal conductivity of graphene 7

Supplementary Note 5. Molecular dynamics simulation of the thermal conductivity of graphene 8

Supplementary Note 6. Mechanism for the enhancement in the Seebeck coefficient of graphene 13

Supplementary Note 7. First principle density functional theory simulation of the electron structure of graphene 14

Supplementary Note 8. Effect of EM interference on graphene 16

Supplementary Note 9. Electric energy output by ordered nanoporous graphene 17

Supplementary Note 10. Effect of thickness and number of ordered nanoporous graphene strips on device output power 18

Supplementary Note 11. Calculation of the overall EM–electricity conversion of ordered nanoporous graphene-based devices 19

Supplementary Figures 20

Supplementary Tables 40

Supplementary Table 1. Representative EM frequency ranges for 3G, 4G, and 5G wireless technology. 40

Supplementary Table 2. Permittivity, polarization frequency, and EM dissipation factor of conventional EM dissipation materials. 41

Supplementary Table 3. Thermal conductivity of a variety of modified graphene, 2D materials, and porous carbons. 43

Supplementary Table 4. Dependence of $d_{1\text{-}4}$ and thermal conductivity of a four-layer graphene nanosheet on its shifting distance, $S_{2\text{-}4}$. 44

Supplementary Table 5. Absolute Seebeck coefficient of conventional 2D nanomaterials. 45

Supplementary Table 6. ZT values of convertional thermoelectric materials and graphene 46

Supplementary Table 7. Maximum power density and temperature difference of current electricity generating materials that utilize temperature gradients. 47

Supplementary Table 8. Thermoelectric coefficients of state-of-the-art thermoelectric materials and bilayer graphene with circular nanopores. **48**

Supplementary Table 9. Experimental parameters for the synthesis of ordered porous graphene with different nanopore shapes. **49**

Supplementary References **50**

Supplementary Notes

Supplementary Note 1. Analysis of the low-frequency polarization relaxation of ordered nanoporous graphene

i) Dipole chemical bonds in ordered nanoporous graphene

The permittivity of graphene as a function of the electromagnetic (EM) frequency is shown in **Supplementary Fig. 5**. For pristine graphene with different numbers of layers, ε' and ε'' decrease as the EM wave frequency increases from 1 to 5 GHz. For graphene with ordered nanopores, a sharp decrease in ε' was observed, and within the same frequency region, ε'' exhibits a remarkable permittivity resonance peak. The existence of a resonance peak in ε'' suggests a frequency dispersive behavior, which is caused by a dipole polarization relaxation behavior[1,2]. According to the classic Debye theory, when a dipole polarization relaxation behavior occurs, the plot of ε' as a function of ε'' forms a semicircle, denoted as the Cole–Cole semicircle. The relative complex permittivity can be written as[3]:

$$\varepsilon_r = \varepsilon_\infty + \frac{\varepsilon_s - \varepsilon_\infty}{1 + j2\pi f\tau} = \varepsilon' - j\varepsilon'' \tag{1}$$

where ε_s, ε_∞, and τ are the static permittivity, relative dielectric permittivity at a high frequency limit, and the dipole relaxation time, respectively. After the separation of real and imaginary parts, we obtain[4]:

$$\varepsilon' = \varepsilon_\infty + \frac{\varepsilon_s - \varepsilon_\infty}{1 + (2\pi f)^2\tau^2} \tag{2}$$

$$\varepsilon'' = \varepsilon_\infty + \frac{2\pi f\tau(\varepsilon_s - \varepsilon_\infty)}{1 + (2\pi f)^2\tau^2} \tag{3}$$

Based on Equations (2) and (3), $\varepsilon' - \varepsilon''$ can be written as:

$$(\varepsilon' - \varepsilon_\infty)^2 + (\varepsilon'')^2 = (\varepsilon_s - \varepsilon_\infty)^2 \tag{4}$$

According to Equation (4), each Cole–Cole semicircle corresponds to one Debye relaxation process. As shown in **Supplementary Fig. 7**, the existence of Cole–Cole semicircles in ordered nanoporous graphene suggests a dipole polarization relaxation behavior at low-frequency regions, which was mainly attributed to the polarization of dipoles at the edge of the nanopores. The rearrangement of dipoles at the pore edge with respect to the EM field leads to a dipole polarization relaxation.

To provide insight into the mechanism of low frequency polarization behavior, we analyzed the crystal structure of graphene. As shown in **Supplementary Fig. 8**, Fourier transform infrared spectroscopy (FT-IR), and X-ray photoelectron spectroscopy (XPS) spectra demonstrate that the dipoles in the ordered nanoporous graphene correspond to –C–OH, –C–O, and –C=O bonds.

ii) Analysis of the polarization frequency of ordered nanoporous graphene

Dipole polarization relaxation frequency is affected by the relaxation time (τ) of the dipoles, which is determined by the type and location of dipoles. Typically, dipoles with a large electronegativity difference ($\Delta\eta$) exhibit large dipole moments and thus large τ, which enables the polarization relaxation behavior in response to low-frequency EM waves[5]. In ordered porous graphene, the dipoles at the pore edges mainly consist of –C=O, –C–OH, and –C–O dangling bonds, which have higher electronegativity difference ($\Delta\eta > 1.4$) than other carbon-involved dipoles in element-doped graphene (such as $\Delta\eta = 0.5$ for C–N, $\Delta\eta = 0.3$ for C–P, $\Delta\eta = \sim 0.1$ for C–S, and $\Delta\eta = \sim 0.3$ for C–H). Besides the dipole type, the distribution of dipoles also affects τ. Compared with non-dangling dipole bonds, the dipoles located at the edge of the pores on the graphene exhibit a larger dipole moment and thus an increase in τ, resulting in a dipole polarization relaxation behavior at low EM frequency.

Supplementary Note 2. Polarization behavior of graphene with different structures

i) Effect of nanopore size on the polarization behavior of graphene

In this section, we synthesized a series of graphene with different structures to confirm the low-frequency dipole polarization relaxation behavior of the ordered nanoporous graphene. First, bilayer graphene consisting of distorted pores with diameters > 20 nm were synthesized by coating excess amounts of nanoparticles on the graphene, as shown in **Supplementary Fig. 9a-b**. This obtained graphene, with polydisperse, large nanopores, possesses the same types of dipoles as the ordered nanoporous graphene. As shown in the Raman spectra (**Supplementary Fig. 9c**), the G-band of the bilayer graphene with polydisperse, large nanopores is broader than that of the bilayer graphene with monodisperse, 6 nm-in-diameter nanopores, suggesting a decrease in the crystallinity of the graphene caused by the polydisperse, large nanopores. In addition, the graphene with polydisperse, large nanopores shows polarization in a high-frequency region (~ 11.8 GHz), as shown in **Supplementary Fig. 9d**.

Next, graphene with nanopores with diameters < 3 nm was synthesized using H_2O_2-assisted chemical etching, as shown in **Supplementary Fig. 9e-f**. The dipole types of this graphene with small nanopores were measured to be the same as graphene with monodisperse, 6 nm-in-diameter nanopores, measured using FT-IR (**Supplementary Fig. 9g**). In addition, we note here that the polarization frequency of graphene with nanopores with diameters < 3 nm is located in the medium frequency region (~ 6.8 GHz), which was still larger than that of graphene with monodisperse 6 nm-in-diameter nanopores, as shown in **Supplementary Fig. 9h**.

ii) Effect of dipole type on the polarization behavior of graphene

In this section, we altered the dipole types of graphene using N- and S-doping (**Supplementary Fig. 9i-j**). As confirmed in FT-IR (**Supplementary Fig. 9k**), C–N and C–S dipoles were formed in N- and S-doped graphene, respectively. In addition, the polarization peaks of both N- and S-doped graphene were located in the high-frequency region (> 8.9 GHz), which is consistent with previous studies (**Supplementary Fig. 9l**)[6,7]. We note here that the polarization frequencies of current EM absorbing composite materials based on graphene lie in the medium- to high-frequency regions, as summarized in **Supplementary Table 2**.

Supplementary Note 3. Effect of pore structure on the polarization intensity of graphene

We hypothesize that the high polarization intensity of circularly porous graphene was due to: (i) the concentration of dipoles and (ii) the pore shapes.

i) Effect of dipole concentration on the polarization of graphene

Considering the fact that all dipoles contain oxygen, we used the oxygen concentration to quantify the concentration of the dipoles in the nanoporous graphene. As shown in XPS, the oxygen concentration in the bilayer graphene with circular, square, and hexagonal nanopores was 13.1 wt %, 15.4 wt %, and 4.9 wt %, respectively. We note here that graphene with hexagonal pores has the lowest oxygen content due to the relatively large pore sizes (16 ± 2 nm) compared with graphene with circular (6 ± 1 nm) and square nanopores (7 ± 1 nm).

ii) Effect of pore shape on the polarization of graphene

According to the polarization mechanism, the polarization intensity depends on the angle between the EM field directions and the dipoles' orientation. A maximum EM energy is required to rotate the dipoles when the EM field and the dipole moment are in parallel. Since the dipoles were located at the pore edges, the intrinsic symmetry of the nanopores affects the dipole polarization relaxation with respect to the direction of the incident EM wave. For circular nanopores, the polarization of the graphene was measured to be independent of the direction of the incident EM wave. For square and hexagonal nanopores, the polarization of the graphene varies as the direction of the incident EM waves changes, according to our previous studies[8].

Supplementary Note 4. Calculation of lattice and electron thermal conductivity of graphene

To provide insight into the reduction in the κ_T of ordered porous graphene, the lattice (κ_L) and electron thermal conductivity (κ_e) can be calculated based on the Widemann–Franz relationship[9]:

$$\kappa_T = \kappa_e + \kappa_L \quad (5)$$

$$\kappa_e = \sigma \times L \times T \quad (6)$$

where L presents the Lorenz number that correlates with S:

$$L = 1.5 + ex(-\frac{|S|}{116}) \quad (7)$$

Supplementary Note 5. Molecular dynamics simulation of the thermal conductivity of graphene

In this section, we performed molecular dynamics simulations to study the effect of the pore structure (e.g., pore size, and staggered porous structure) and the number of graphene layers on the κ_T of porous graphene. We used the Tersoff potential with an optimized parameter set to model graphene, and we applied a reverse non-equilibrium molecular dynamics simulation to calculate the κ_T of graphene based on Fourier's law of thermal conduction[10]:

$$J = -\kappa_T \nabla T \quad (8)$$

where J is the heat flux and ∇T is the temperature gradient in the heat flux direction. In our modelling, the initial atomic configuration was equilibrated at T = 300 K in a canonical ensemble (NVT) for 20 ps with a time step Δt = 1 fs.

After the system reached equilibrium (**Supplementary Fig. 14a**), the system is switched to a microcanonical ensemble (NVE) for energy conservation. To create a temperature gradient along the graphene direction, the atoms located at the left end (in a region with a width of 10 Å; highlighted in red) and the right end (in a region with a width of 10 Å; highlighted in blue) were kept at T = 350 K (heat source) and T = 300 K (heat sink), respectively, by coupling the carbons with a Nosé–Hoover thermostat. In the NVE ensemble, the simulation was conducted for 100 ps to allow the system to reach a steady state after applying a temperature gradient along the x direction. After forming the stable temperature gradient in the sample, the simulation was conducted for another 50 ps to obtain the heat flux and the time-averaged temperature profile. To calculate the heat flux, the accumulative energy changes in both the heat source and sink regions were recorded as a function of simulation time, and the energy change rate represents the heat flux. To obtain the temperature profile, we divided the entire system into a series of thin slabs with widths of 10 Å along the x direction, and the temperature of each slab was calculated based on the kinetic energy of all the atoms within the slab.

i) Effect of pore size on the thermal conductivity of graphene

Supplementary Fig. 14b shows the atomic configurations of monolayer graphene nanosheets. In each graphene nanosheet, the pores were arranged into three rows that are separated by a distance, χ, in the x-direction, and the adjacent rows were shifted by a distance equivalent to the size, d, of the pore with respect to each other in the y direction. The width of the nanoribbons was defined as the edge-to-edge distance between the two nearest neighboring pores (denoted as ω). The dimensions of the monolayer graphene nanosheet is set as $(3\chi + 30) \times 2\chi \times 3.4$ Å^3, in which $\chi = (d + \omega)$. A periodic boundary condition was applied in the y direction, while a free boundary condition was applied in the z direction. Both ends of the graphene nanosheets are fixed during the simulation by freezing the carbon atoms in two thin slabs with a thickness of ~ 5 Å (highlighted in gray in **Supplementary Fig. 14b**).

Supplementary Fig. 14c-e plots the effect of the pore size and the width ω on the ratio of the

κ_T of monolayer porous graphene nanosheet to the κ_T of nonporous monolayer graphene (defined as the κ_T ratio). We observed that the κ_T ratio decreases with an increase in the pore size and increases with an increase in the nanoribbon width, which is consistent with past simulation studies of graphene nanomesh[11] and graphene kirigami[12]. These results reveal that the presence of ordered nanopores on the graphene surface leads to a significant reduction of the κ_T of graphene (the ratio of κ_T is < 5%), which is consistent with our experimental measurements. Furthermore, we observe that an increase in the pore size decreases the κ_T ratio for the same pore size. For example, the ratio of κ_T of graphene with circular nanopores decreases from 4.8% to 3.1%, with an increase in the pore diameter from 2 to 5 nm.

ii) Effect of pore density on the thermal conductivity of graphene

In addition to the pore diameter, we studied the effect of pore density on the κ_T of graphene by varying ω. In our simulation, the pore diameter was set at 4 nm. As shown in **Supplementary Fig. 14f-h**, a decrease in ω, equivalent to an increase in the pore density, reduces the κ_T of ordered porous graphene. For example, the ratio of κ_T decreases from ~ 8.2 % to ~ 1.8 %, with a decrease in ω from 2.5 to 1.0 nm.

In a nanoporous graphene nanosheet, the main thermal conduction paths are the nanoribbons between two adjacent nanopores and parallel to the heat transport direction. Past molecular dynamics simulations on the nanoribbon of two-dimensional nanomaterials have shown that the edges of the nanoribbons significantly affect their κ_T because of the edge localization and boundary scattering effects, both of which arise from the mismatch (or weak coupling) of the phonon density of states of the carbon atoms at the nanoribbon edge and the density of states of the atoms in the nanoribbon (away from the edge)[13,14,15]. We hypothesize here that the reduction in the κ_T of ordered porous graphene is attributed to two mechanisms: (i) weak coupling of phonons at the pore edge and (ii) backscattering of phonons at the pore edges. The first mechanism leads to an enhanced three-phonon Umklapp scattering in parallel nanoribbons, while the second mechanism significantly elongates the phonon transport pathway due to the existence of the perpendicular (or close to perpendicular) nanoribbon[16].

To provide insight into the reduction in the κ_T of ordered nanoporous graphene, we calculated the phonon density of states of carbon atoms in graphene nanoribbon with armchair and zigzag edges. As shown in **Fig. 3f** of the main text and **Supplementary Fig. 15**, a clear difference between the phonon density of states of carbon atoms at the edge of the nanoribbon and carbon atoms in the nanoribbon (away from the edge) was observed in both types of graphene nanoribbons. Particularly, in the frequency range between 50 and 65 THz, the phonon density of states of carbon atoms in the nanoribbons is negligible, while a peak exists in the phonon density of states of the C atoms at the pore edges. These calculation results suggest that phonons in this frequency range are localized at the edges of graphene nanoribbons and are not able to transport energy as efficiently as the delocalized phonons in the graphene nanoribbons, which

is consistent with previous simulation studies that the coupling degree of phonons in graphene nanoribbons is only 0.2 (coupling degree of completely coupled phonons is 1.0).

In addition to the weak coupling of phonons localized at the edge, backscattering of phonons at the pore edges caused by the mismatch in phonon density of states with frequencies lower than 50 THz also contributes to the decrease in the κ_T of porous graphene. To provide more evidence, we calculated the time-averaged spatial distribution of heat flux on each atom in the ordered nanoporous graphene using vector arrows after establishing a stable temperature gradient in the graphene. The vector arrows show the transport of heat flux in the porous graphene nanosheets and the associated heat flux transport pathways and the phonon scattering at the pore edges. The atomic heat flux is described using the following equation[17]:

$$J_i = \varepsilon_i \boldsymbol{v_i} - \boldsymbol{S_i} v_i \quad (9)$$

where ε_i, $\boldsymbol{v}_i$, and $\boldsymbol{S}_i$ are the atomic energy, the velocity vector, and the stress tensor of atom i, respectively. As shown in **Supplementary Fig. 16**, during the propagation of a phonon from the heat source to the heat sink, it is backscattered to change its propagation direction when it encounters a pore edge. This backscattering effect largely reduces the mean free path of phonons and significantly increases the transport pathways of phonons (indicted by the black arrows in **Supplementary Fig. 16**) in nanoporous graphene, resulting in a significant decrease in the κ_T. We comment here that for both the weak coupling and the backscattering of phonons at the pore edges, their effects on κ_T are related to the edge-to-surface area ratio of the porous graphene nanosheet. Our simulation indicates that a high edge-to-surface area ratio leads to a high probability for phonons to be trapped and backscattered at the pore edges.

iii) Effect of the number of graphene layers on the thermal conductivity of graphene

Here we studied the effect of the number of graphene layers on the κ_T of the ordered porous graphene. In our simulation, the pore size was kept at 4 nm, the width of the nanoribbons was set at 2.5 nm, and the interlayer distance was set to be 0.34 nm, which is equal to the interlayer distance of bulk graphite. Inspection of **Supplementary Fig. 17** reveals that κ_T decreases with an increase in the number of graphene layers, which is consistent with previous studies on the κ_T of multilayer graphene nanosheets and graphene nanoribbons[18]. It has been experimentally and computationally demonstrated that the cross-plane phonon resonance effect (also known as cross-plane phonon coupling) alters the basal plane κ_T of multilayer graphene nanoribbons, leading to an enhancement in the three-phonon Umklapp scattering[19].

iv) Effect of the staggered pore structure on the thermal conductivity of graphene

We note here that in our simulation described above, the graphene nanosheets are completely overlapped with each other in the multilayer graphene. However, in our experiments, we observed a staggered porous structure in multilayer graphene with nanopores. Therefore, we simulated the effect of the overlap ratio of graphene layers in four-layer graphene nanosheets

with square pores. The overlap ratio was tuned by shifting the second and fourth graphene layers along the y-direction, as shown in **Supplementary Fig. 17a**. For simplicity, we utilized the distance between the center of mass of the first and fourth graphene layers along the graphene thickness direction (z-axis) as the overlap ratio. We note here that it is difficult to control and determine the overlap ratio of multilayer nanoporous graphene in our experiments. As shown in **Supplementary Fig. 18b**, we observed the overlap ratio of the graphene layers has a significant impact on the κ_T of multilayer graphene. We hypothesize here that the shifting of the second and fourth graphene layers reduces the screening effect between adjacent graphene layers and reduces the interlayer spacing between graphene, which is consistent with our experimental observations shown in **Supplementary Fig. 20**.

Furthermore, we note here that the attraction across graphene layers imposes a compression on the whole nanosheet, particularly in areas where the graphene nanoribbons have a high overlap ratio. For multilayer nanoporous graphene nanosheets, the smaller the overlap ratio of two adjacent graphene layers is, the more intense the compression the constitutive graphene layers impose on the whole nanosheet. To provide theoretical support, we simulated the distance between the center of mass of the first and the fourth layer of the four-layer porous graphene nanosheet ($d_{1\text{-}4}$) as a function of the shifting distance of the second and the fourth layer ($S_{2\text{-}4}$). As shown in **Supplementary Table 4**, for all $S_{2\text{-}4}$ ranging from 0 to 100 Å, $d_{1\text{-}4}$ is ~ 1 Å, which is smaller than that of perfectly overlapped graphene nanosheets, except for $S_{2\text{-}4}$ = 60 Å. We note here that at $S_{2\text{-}4}$ = 60 Å, the shifting distance is equal to the periodicity of the square pores, and the graphene layers are fully overlapped in the same way as $S_{2\text{-}4}$= 0 Å. In addition, we have simulated a five-layer graphene nanosheet with square nanopores. We calculated the difference in distances between the center of mass of the first and the fifth layer ($d_{1\text{-}5}$) of perfectly overlapped graphene nanosheets and partially overlapped graphene to be ~ 1.5 Å. This simulation result is in agreement with our experimental measurement that the interlayer spacing between nanoporous graphene with limited numbers of layers was measured to be ~ 3.2 Å, and it decreases to ~ 2.9 Å nm as the number of layers increases.

Next, we calculated the κ_T of the four-layer nanoporous graphene nanosheet as a function of $d_{1\text{-}4}$. As shown in **Supplementary Table 4**, the κ_T decreases with a decrease in $d_{1\text{-}4}$. We hypothesize that this trend can be attributed to an enhanced ross-plane resonance effect and an edge-induced phonon scattering effect in partially overlapped nanoporous graphene. To quantify the enhancement of the edge phonon scattering, the coupling of the phonon spectrum ($P_1(\omega)$) of atoms at the edges of the graphene nanoribbon and the phonon spectrum ($P_2(\omega)$) of atoms in the middle of the graphene nanoribbon is calculated as[20]:

$$\text{Phonon coupling } = \left(\int \sqrt{P_1(\omega)P_2(\omega)}\,\mathrm{d}\omega\right)^2 / \left(\int P_1(\omega)\mathrm{d}\,\omega \int P_2\,(\omega)\mathrm{d}\omega\right) \quad (10)$$

When the graphene nanoribbons are partially overlapped, the calculated phonon coupling

factor of the graphene nanoribbons with armchair edges is significantly reduced compared with perfectly overlapped graphene nanoribbons, which is similar to that of graphene with zigzag edges, as shown in **Supplementary Fig. 18b** and **Fig 3g** of the main text. A reduction in the phonon coupling factor suggests that the overlapped area of adjacent graphene nanosheets decreases, resulting in an enhanced backscattering of phonons at the pore edges and a decrease in κ_T of the graphene nanosheet.

To further validate the second mechanism and obtain the dependence of the thermal conductivity on the overlap ratio, we simulated the κ_T of a monolayer graphene nanoribbon with periodic pores covered by a defect-free, nonporous graphene nanoribbon and the overlap ratio between the porous graphene and the nonporous graphene is precisely tuned by adjusting the width of the top nonporous graphene, as schemed in **Supplementary Fig. 19a**. The overlap ratio is 0 and 1 for no overlapping and complete overlapping of the pores by the covering graphene, respectively. Inspection of **Supplementary Fig. 19b** reveals that as the width of the nonporous graphene increases, the κ_T of the porous graphene increases due to the coupling between the atoms at the pore edge of the porous graphene and the atoms in the defect-free, nonporous graphene.

Supplementary Note 6. Mechanism for the enhancement in the Seebeck coefficient of graphene

In general, the absolute value of the Seebeck coefficient |*S*| is affected by the carrier concentration, effective mass, and electronic structure.

i) Effect of carrier concentration on the Seebeck coefficient of graphene

We measured the carrier concentration (n) as a function of the temperature via a Hall carrier measurement. As shown in **Supplementary Fig. 11e-h**, compared with nonporous graphene, the presence of pores reduces n by 37.9–57.6 %, which contributes to the enhancement in |*S*| of porous graphene.

ii) Effect of the effective mass on the Seebeck coefficient of graphene

In general, the effective mass (m*) is proportional to |*S*| and can be qualitatively determined by its inverse proportional relationship to the mobility (μ_H)[21]. The μ_H can be calculated as[22]:

$$\mu_H = \sigma/ne \tag{11}$$

where σ is the electric conductivity, and e is the electronic charge (1.6×10^{-19} C). The calculated μ_H of both the porous and nonporous graphene is shown in **Supplementary Fig. 11h-k**. We found that the creation of nanopores on the graphene surface will significantly reduce the μ_H of graphene due to the decrease in the electron transport path. For example, the μ_H of bilayer graphene with circular nanopores is 55.2–72.7 $cm^2/V{\cdot}s$, which is 56.0–72.3 % of that of bilayer nonporous graphene. The decrease in the μ_H value indicates an increase in m*, which is beneficial to the absolute |*S*| value.

iii) Effect of electronic structure on the Seebeck coefficient of graphene

The |*S*| value of graphene can also be tuned via the electronic structure of graphene, including the Fermi surface, energy band structure, and bandgap value[23]. We have discussed the role of the Fermi surface and energy band structure in the section of the density functional theory simulation (see **Supplementary Note 7**). Nonporous graphene has a zero bandgap value and a negligible |*S*|. Here we sought to study the effect of nanopores on the bandgap value of ordered porous graphene.

Overall, the enhanced |*S*| of graphene with ordered nanometer-sized pores is due to a reduction in n, an increase in m*, and the open bandgap structure, although the quantitative relationship between the bandgap value and |*S*| has yet to be understood.

Supplementary Note 7. First principle density functional theory simulation of the electron structure of graphene

In our work, we used first principles density functional theory to simulate the band structure of graphene. Our simulation includes crystal model design and optimization, the building of a static self-consistent field, and a density of states calculation. All calculation steps are completed in VASP-6.1.0[24], combined with the Perdew–Burke–Ernzerhof (PBE) functional[25,26] under the generalized gradient approximation[27]. The frame of density functional theory with D3 dispersion correction[28] is combined with the projector augmented wave[29], and the plane wave cut-off energy was set to 500 eV. The K point adopts the density of 3 × 3 × 1. The finite displacement method combined with phononpy-2.11[30] was used to calculate the phonon structure of graphene. Here we studied graphene with three structure models: i) monolayer graphene with a single 1.2 nm-size circular pore at the center of the surface; ii) bilayer graphene with a 1.2 nm-diameter circular pore on each graphene layer that have different overlap ratios (complete overlap and 1/3 overlap); and iii) trilayer graphene with a circular pore on each graphene layer that has a 1/3 overlap ratio with respect to the other two layers. The carbon atoms at the pore edge were saturated by –H, –OH, =O and –O–.

i) Effect of porous structure on the Seebeck coefficient of graphene

To reveal the effect of porous structure on $|S|$ of graphene, we calculated the electron structure of graphene with circular nanopores. To exclude the interference of a staggered porous structure, we simulated monolayer porous graphene. As shown in **Supplementary Fig. 28**, monolayer graphene with circular pores shows a similar distribution of density of states, suggesting a similar electron structure in these two types of monolayer graphene. However, we found that the first phonon curve of monolayer graphene with circular nanopores was located at a low frequency. We note here that in monolayer graphene with circular pores, the inflection point of the low frequency and high frequency acoustic branch phonons form a parabolic phonon pocket, which greatly enhances the Seebeck effect of monolayer graphene with circular pores.

ii) Effect of the number of graphene layers on the electron structure of graphene

To study the effect of the number of layers, we compared the electron structure of bilayer and trilayer graphene with circular nanopores at the same overlap ratio (1/3). Both graphene structures shifted the Dirac points away from their original position (Dirac points of a pristine, nonporous graphene are located on G, M, and K) to the regions between G–M and K–G, and split the Dirac points, resulting in an open bandgap structure and the breaking of the Fermi surface (the Dirac trap effect). We note that although the bandgap value of trilayer graphene with circular nanopores is ~ 0.25 eV, much higher than that of bilayer porous graphene, suggesting a strong electron confinement ability of trilayer nanoporous graphene. However, the calculated projection density of states of trilayer nanoporous graphene shows that the

intensity of the density of states near zero density of states is much weaker than that of its bilayer counterpart, suggesting a weak interaction intensity between phonons and electrons (phonon drag effect). As a result, $|S|$ of trilayer graphene with circular nanopores is lower than that of its bilayer counterpart.

iii) Effect of pore overlap ratio on the electron structure of graphene

We computed the electron structures of bilayer porous graphene with different overlap ratios. We observed a splitting of the Dirac points in all the bilayer porous graphene, and bilayer porous graphene with an overlap ratio of 1/3 has the largest bandgap value (0.13 eV). This result suggests that bilayer graphene with completely overlapped pores has a weak ability in electron confinement. Furthermore, there was no significant van Hoff singularity point nearing the zero density of states in bilayer graphene with completely overlapped pores, suggesting a weak phonon drag effect.

Supplementary Note 8. Effect of EM interference on graphene

Upon EM radiation, the formed temperature gradient and the associated electrical potential drive the movement of electrons from the high temperature end to the low temperature end. In addition to the temperature gradient, the surrounding EM field may deviate from the movement of electrons from the direction of the temperature gradient in the graphene. As a result, it may decrease the effective electric potential. To quantify the effect of EM interference, we compared the electrical potential of ordered nanoporous graphene generated by EM radiation with that generated by applying an equivalent temperature gradient. As shown in **Supplementary Fig. 29a**, the ratio of electric potential generated by EM radiation and that generated by applying a temperature gradient is 86.7–96.3 %, indicating that the impact of EM field interference is not severe in ordered porous graphene. In contrast, this ratio is 38.5–45.1 % in nonporous graphene (**Supplementary Fig. 29b**), suggesting the presence of a remarkable EM field interference in nonporous graphene.

We hypothesize that the EM interference intensity is caused by two factors: i) low frequency polarization behavior, and ii) ordered nanopore structure.

i) Effect of low frequency polarization behavior

In general, the types of EM dissipation include polarization relaxation loss and conductive loss. In nonporous graphene, the EM dissipation is dominated by conductive loss caused by the non-directional movement of carriers, during which the resistance or collision of the moving carriers is dissipated into heat. However, in ordered porous graphene, the dominating EM dissipation type is the polarization relaxation loss, which relies on the dipole relaxation to dissipate EM energy into heat, rather than conductive loss. The presence of nanopores reduces the non-directional movement of carriers and thus efficiently decreases the polarization relaxation loss.

ii) Effect of the ordered nanoporous structure

Ordered porous graphene consists of carbon atom regions among nanopores, which are equivalent to graphene nanoribbons. We hypothesize that the electrons can directionally transport in the regions among the nanopores, which decreases the random movement of electrons and thus conductive loss, as shown in **Supplementary Fig. 30**.

Supplementary Note 9. Electric energy output by ordered nanoporous graphene

i) Internal resistance of ordered nanoporous graphene

Here we measured the EM wave radiation-induced electric energy output by ordered nanoporous graphene with a loaded resistance ranging from 0–150 Ω. As shown in **Fig. 5b**, with the same loaded resistance, the output voltage (V_L) reaches a maximum at 180 s of EM radiation. Based on V_L, we calculated the output power as a function of the resistance, as shown in **Fig. 5c**. We found the maximum output power to be ~ 1.51 mW with a resistance of 60 Ω after an EM radiation of 180 s. We note here that the graphene-based device has an internal resistance of 60 Ω (10 Ω for each graphene strip).

ii) Electric power output as a function of time

Based on the results shown in our previous section (maximum V_L at 180 s of EM radiation), we measured ΔT as a function of time during 180 s of EM radiation. We found that ΔT increases in the first 180 s of EM radiation and decreases to zero within 180 s upon the removal of the EM radiation, as shown in **Fig. 5e** of the main text. The time-dependent power output can be calculated as:

$$W_{out} = P_{out} \times t \tag{12}$$

where t is the time, and W_{out} and P_{out} are the output work and output power, respectively. According to Equation 12, the output work of graphene is calculated to be 2.3 mJ within 360 s.

iii) Output work of graphene-based devices at elevated temperatures

Considering the fact that the temperature of the electronic device can increase during usage [31], here we sought to investigate the effect of the temperature on W_{out} of the graphene-based devices. As shown in **Supplementary Fig. 32**, W_{out} of the graphene-based devices remains almost constant over a wide temperature range from 10 to 70 °C.

Supplementary Note 10. Effect of thickness and number of ordered nanoporous graphene strips on device output power

With a constant incoming EM power, we hypothesize that the ordered nanoporous graphene-based device's output power depends on (i) the thickness and (ii) the number of the nanoporous graphene strip.

i) Effect of the thickness of the strips on the device's output power

As shown in **Supplementary Fig. 31a**, as the thickness of the ordered nanoporous graphene strip increases, the device output power first increases and then decreases and the maximum output power reaches ~ 3.1 μW, almost three times that of a 10 μm-thick ordered nanoporous graphene strip. According to the EM shielding mechanism, with an increase in the EM absorbing material thickness, the EM wave dissipation path increases and the inverse radiation decreases, which is consistent with our experimental observation that the EM absorption increases monotonically from 11% to 29% as the thickness increases from 10 μm to 50 μm (**Supplementary Fig. 31a**). The decrease in output power with an increase in thickness from 25 μm to 50 μm is attributed to the increase in the internal resistance of the device.

ii) Effect of the number of strips on the device's output power

As illustrated in **Supplementary Fig. 31b**, the output power of the device increases from 1.5 μW to 10.5 μW as the number of ordered nanoporous graphene strips increases from 6 to 30. This increase in output power is due to the increase in device volume as the number of ordered nanoporous graphene strips increases, leading to an increase in the amount of absorbed EM waves. These results suggest that the device output power can be increased by increasing the surface area and the number of ordered nanoporous graphene strips.

Supplementary Note 11. Calculation of the overall EM–electricity conversion of ordered nanoporous graphene-based devices

The overall EM–electricity conversion (x) of our ordered porous graphene-based device can be calculated as:

$$x = \frac{W_{\text{out}}}{\frac{V_{\text{device}}}{V_{\text{chamber}}} W_{\text{source}} \alpha} \tag{13}$$

where W_{out} is the output work of the graphene device (0.23 mJ during 180 s of EM radiation), W_{source} is the output work of the EM generator (100 W), α is the attenuation degree, and V_{device} and V_{chamber} represent the volume of the graphene device (six graphene strips; each strip is 25 mm × 5 mm × 0.01 mm) and EM radiation chamber (447 mm × 247 mm × 281 mm), respectively. It is well established that the GHz-frequency EM wave attenuation is negligible in a chamber with these dimensions ($\alpha \approx 1$), which gives rise to an almost constant EM intensity in the chamber[31, 32]. Therefore, the fraction of the EM waves exposed to the graphene device can be calculated by the volume fraction of the graphene device relative to the chamber volume. As a result, the overall EM–electricity conversion of the bilayer porous graphene-based device was calculated to be 5.6 %.

Supplementary Figures

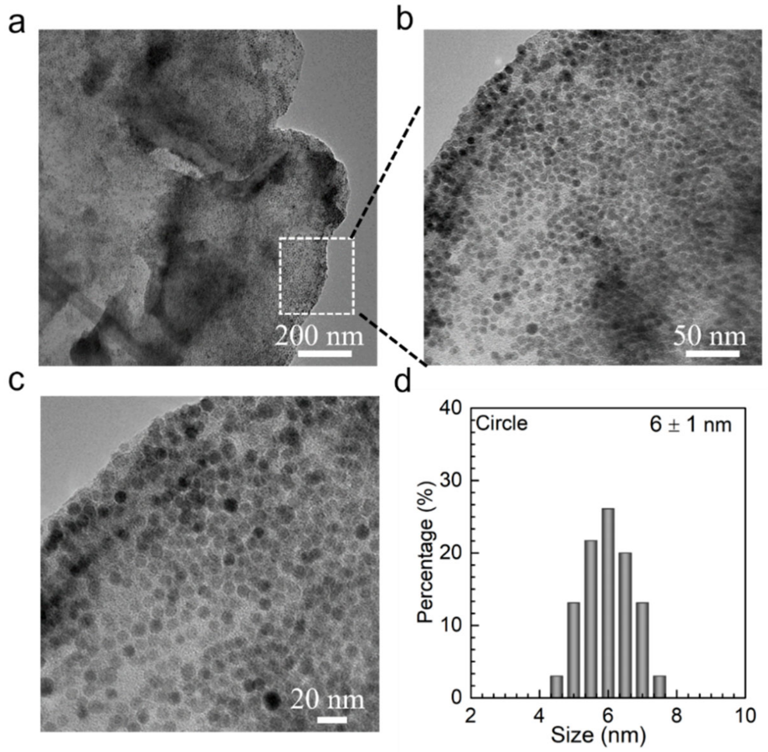

Supplementary Fig. 1 - *In-situ* growth of spherical Fe_3O_4 nanoparticles on graphene. (a-c) Representative TEM images of spherical Fe_3O_4 nanoparticles on graphene. (d) Diameter distribution of Fe_3O_4 nanoparticles grown on graphene.

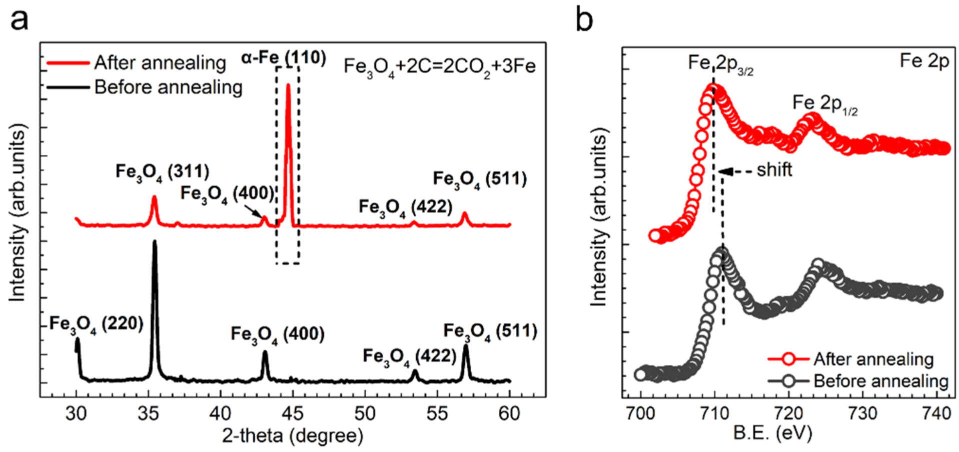

Supplementary Fig. 2 - Crystal phase and composition evolution of Fe_3O_4 nanoparticle-coated graphene upon annealing. (a) X-ray diffraction (XRD) patterns and (b) *Fe 2p* XPS spectrum of Fe_3O_4-coated graphene before and after annealing at 900 ºC. The appearance of a diffraction peak at $2\theta = 44.4^o$ in the annealed Fe_3O_4-coated graphene is attributed to the (110) crystal plane of α-Fe. The shifting of the *Fe* $2p_{3/2}$ peak to low binding energy upon annealing is attributed to the reduction of Fe by carbonization.

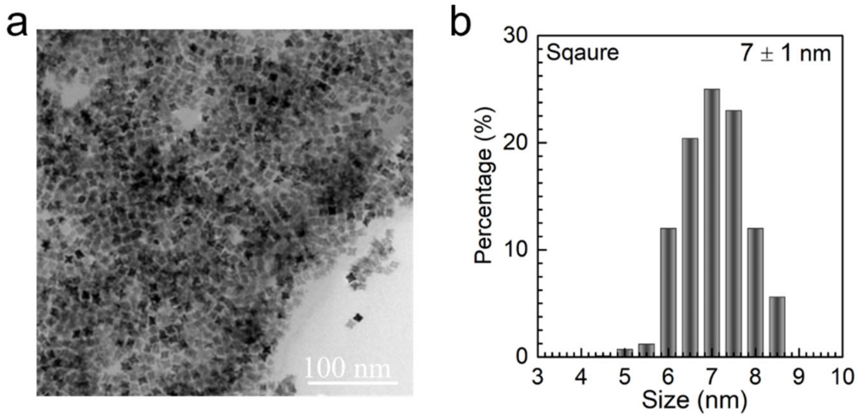

Supplementary Fig. 3 - *In-situ* growth of cubic Fe_3O_4 nanoparticles on graphene. (a) Representative TEM images of cubic Fe_3O_4 nanoparticles on graphene. (b) Size distribution of cubic Fe_3O_4 nanoparticles grown on graphene.

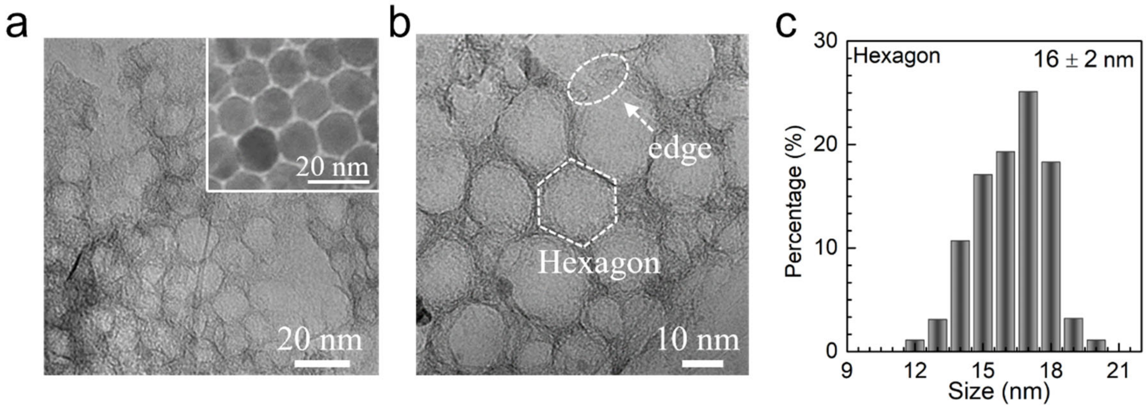

Supplementary Fig. 4 - Morphology of nanoporous graphene templated by hexagonal Fe_3O_4 nanoparticles. (a, b) Representative TEM images of nanoporous graphene templated by hexagonal Fe_3O_4 nanoparticles. Inset in (a) is a TEM image of hexagonal Fe_3O_4 nanoparticles. (c) Size distribution of hexagonal Fe_3O_4 nanoparticles grown on graphene.

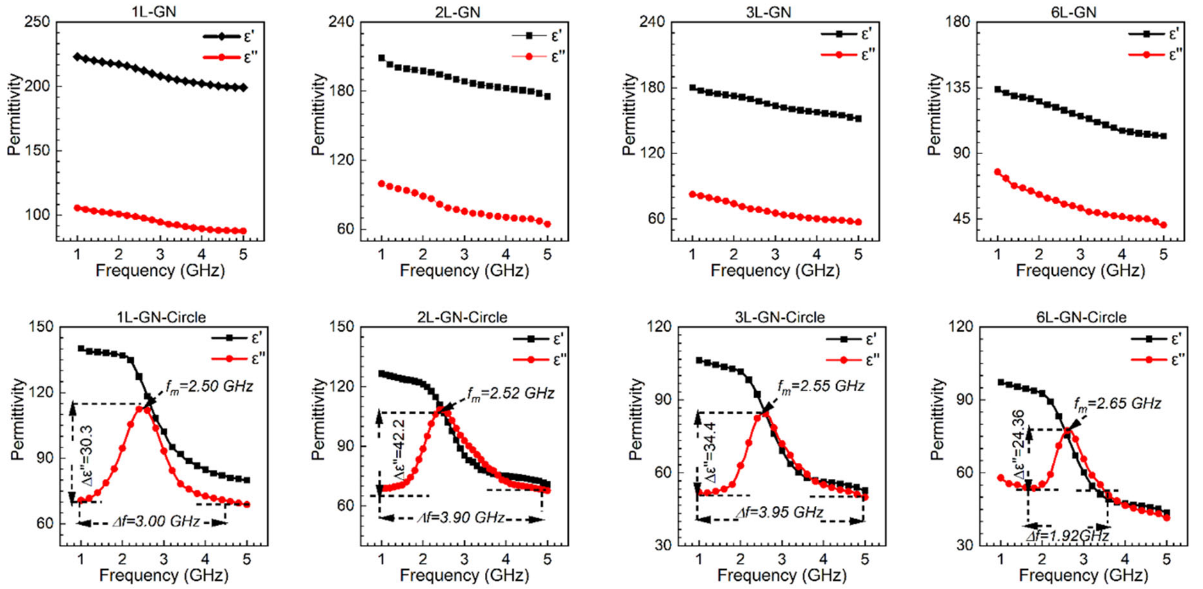

Supplementary Fig. 5 - Frequency-dependent permittivity of graphene with circle nanopore and different numbers of layers: ε' and ε'' represent the real part and imaginary part of the permittivity, respectively. '1L', '2L', '3L' and '6L' represent 1, 2, 3 and 6 layers, respectively. 'GN' and 'GN-Circle' represent pristine graphene and graphene with circular nanopores, respectively.

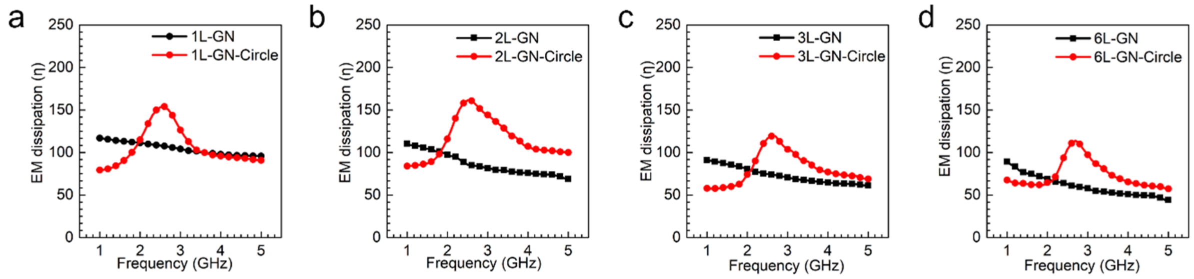

Supplementary Fig. 6 - Calculated EM dissipation factor of graphene with circle nanopore and different numbers of layers: EM frequency-dependent EM dissipation factor of graphene with (a) 1, (b) 2, (c) 3, and (d) 6 layers. The presence of nanoporous structures enables efficient EM dissipation within the EM frequency range of 2–5 GHz. '1L', '2L', '3L' and '6L' represent 1, 2, 3 and 6 layers, respectively. 'GN' and 'GN-Circle' represent pristine graphene and graphene with circular nanopores, respectively.

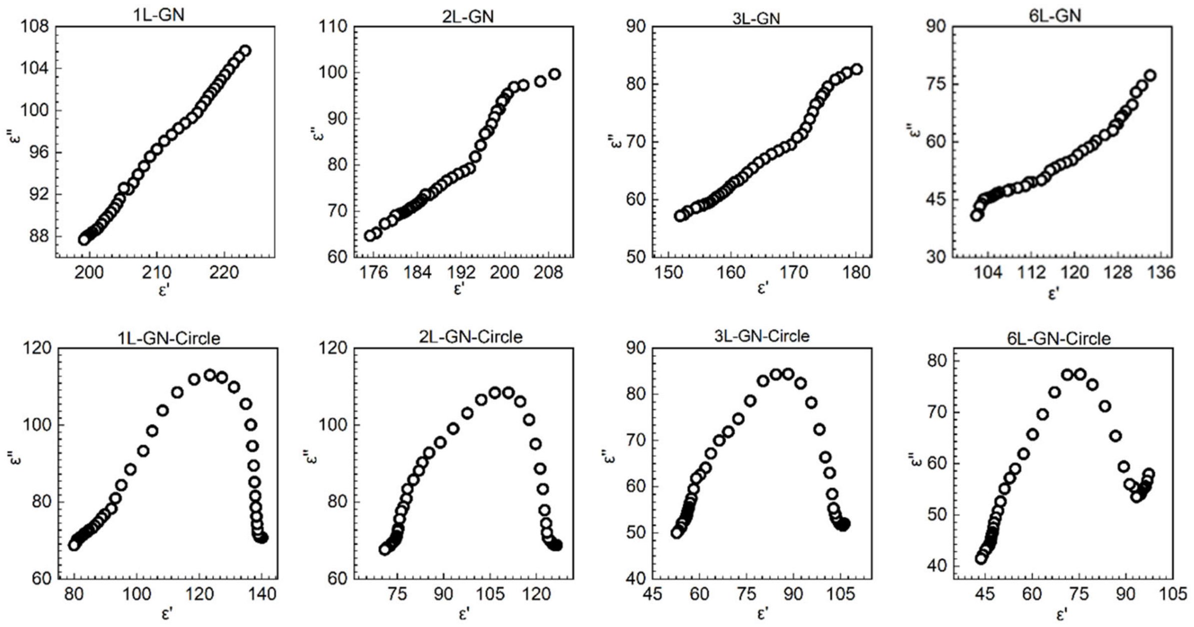

Supplementary Fig. 7 - Polarization relaxation behavior (Cole–Cole curve) of graphene with circle nanopore and different numbers of layers. The Cole–Cole curves were plotted using ε' and ε''. ε' and ε'' represent the real part and imaginary part of the permittivity, respectively. The presence of semi-circles in the Cole–Cole curves suggests the existence of a polarization relaxation behavior in the ordered nanoporous graphene. '1L', '2L', '3L' and '6L' represent 1, 2, 3 and 6 layers, respectively. 'GN' and 'GN-Circle' represent pristine graphene and graphene with circular nanopores, respectively.

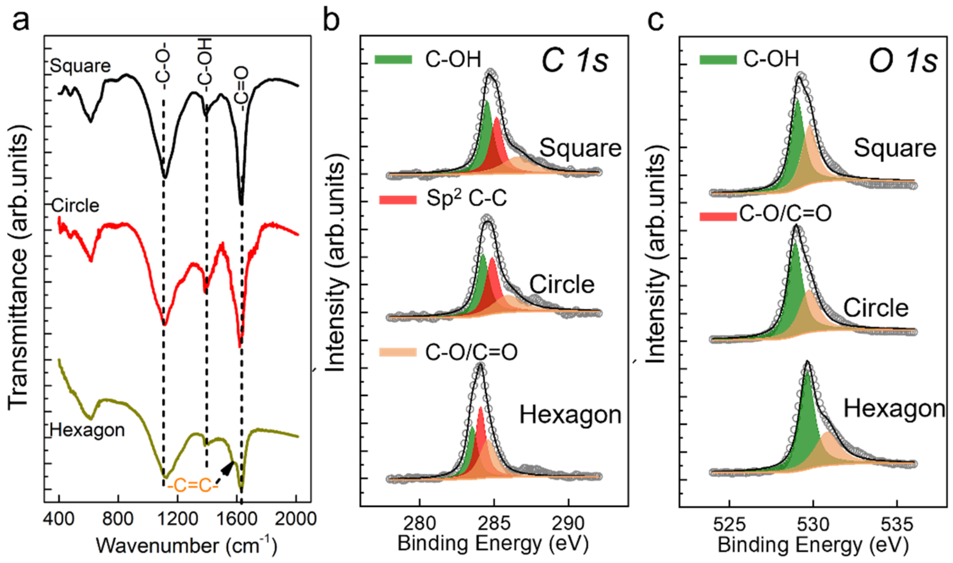

Supplementary Fig. 8 - Analysis of elements and dipole bonds in ordered nanopore graphene. (a) FT-IR spectra of porous bilayer graphene. XPS of (b) *C 1s* and (c) *O 1s* spectra of porous bilayer graphene.

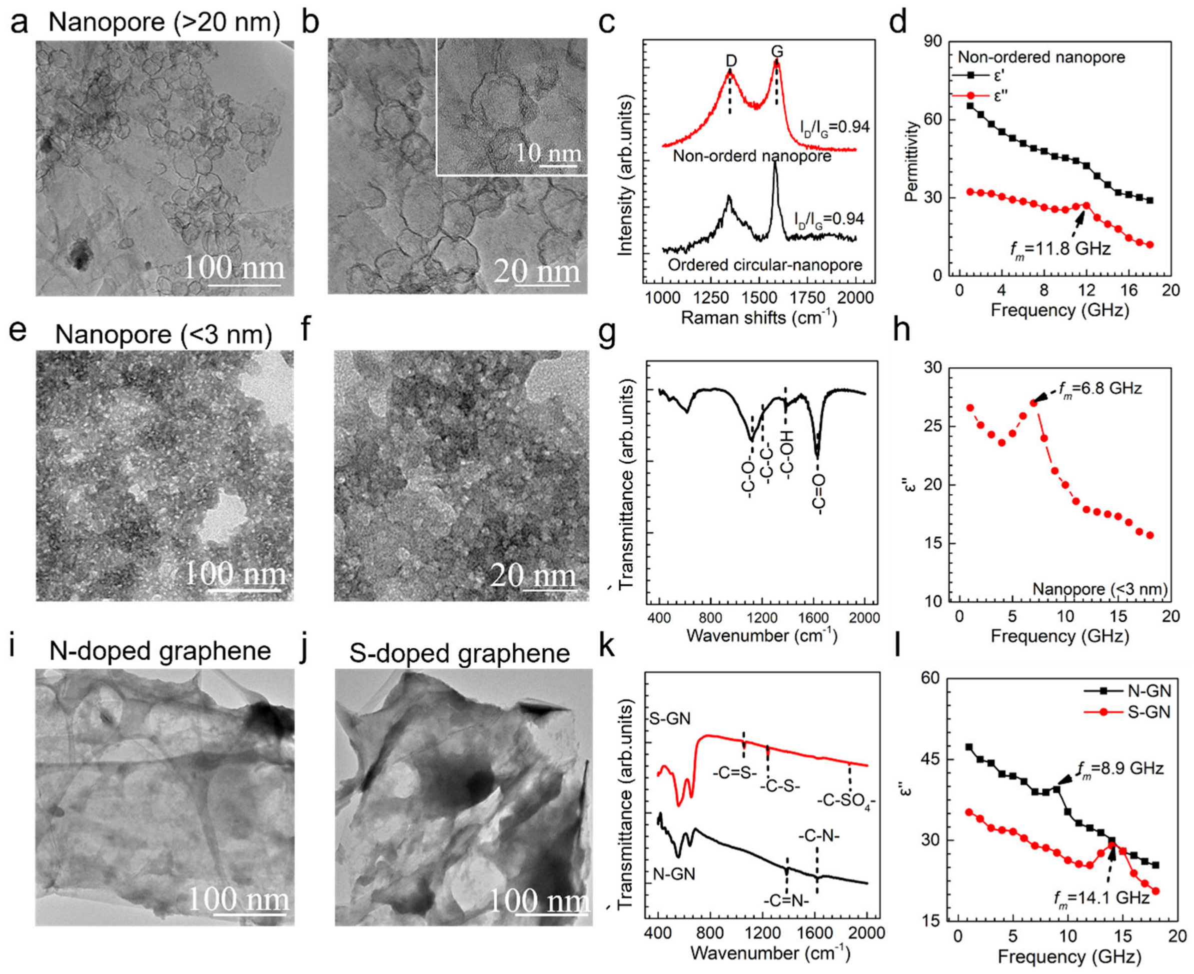

Supplementary Fig. 9 - Dipole relaxation polarization of porous graphene and element-doped graphene. (a, b, e, f, i, j) TEM images, (c) Raman spectra, (g, k) FT-IR spectra and (d, h, l) EM frequency-dependent permittivity of (a-d) graphene with pore sizes exceeding 20 nm, (e-h) graphene with pore sizes less than 3 nm, and (i-l) N or S-doped graphene (N-GN and S-GN, respectively). In (c), two broad peaks centered at 1350 and 1593 cm^{-1} represent to typical D- and G-bands of carbon, respectively. The intensity ratio of the D- and G-bands (I_D/I_G) represents the graphitization level.

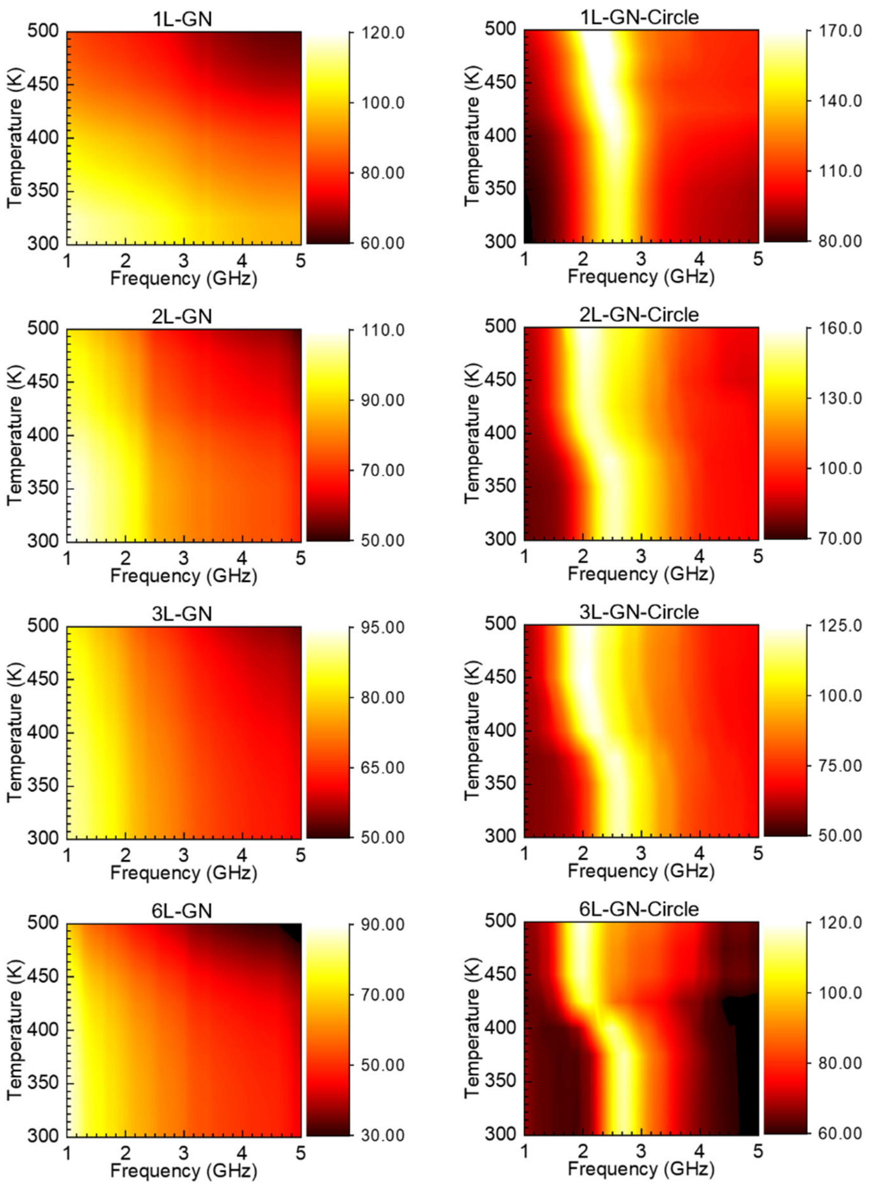

Supplementary Fig. 10 - EM dissipation factor of graphene at evaluated temperatures. Compared with pristine graphene, circular nanoporous graphene exhibits an enhanced EM dissipation at elevated temperatures. ‘1L’, ‘2L’, ‘3L’ and ‘6L’ represent 1, 2, 3 and 6 layers, respectively. ‘GN’ and ‘GN-Circle’ represent pristine graphene and graphene with circular nanopores, respectively.

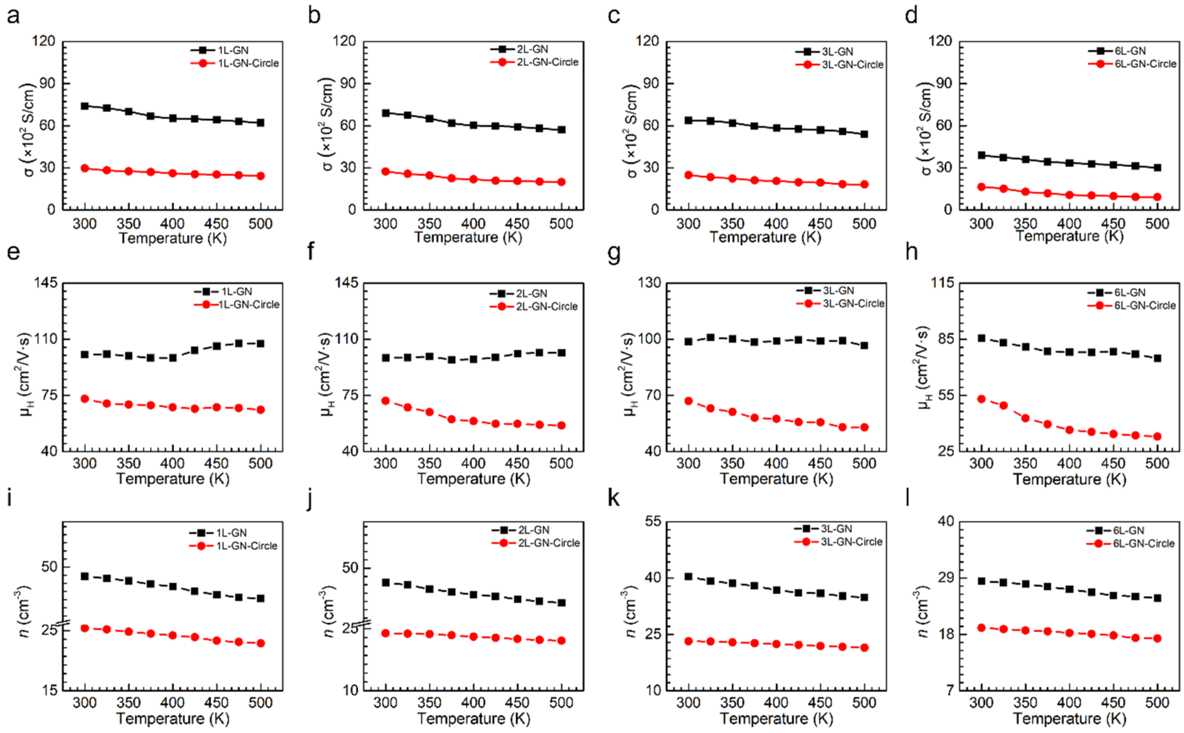

Supplementary Fig. 11 - Hall carrier density and mobility and the electrical conductivity of graphene with circle nanopore and different numbers of layers. (a-d) Hall carrier density (n), (e-h) mobility (μ_H) and (i-l) electrical conductivity (σ) of porous graphene with (a, e, i) 1, (b, f, j) 2, (c, g, k) 3, and (d, h, l) 6 layers as a function of temperature. '1L', '2L', '3L' and '6L' represent 1, 2, 3 and 6 layers, respectively. 'GN' and 'GN-Circle' represent pristine graphene and graphene with circular nanopores, respectively.

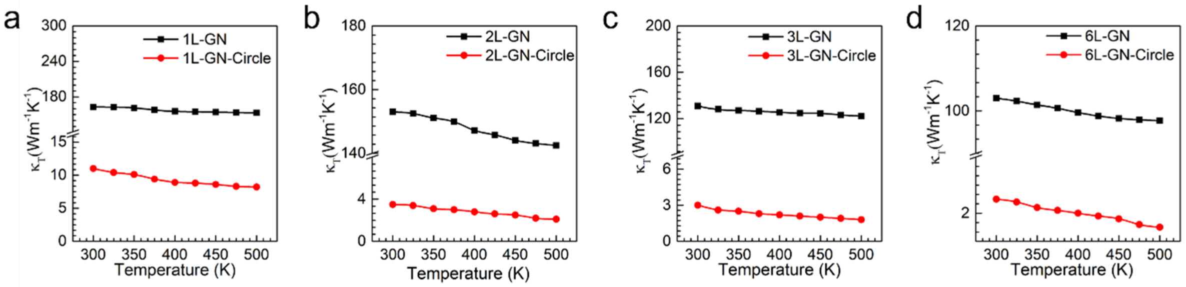

Supplementary Fig. 12 - Thermal conductivity (κ_T) of graphene with circular nanopore and different numbers of layers. (a) 1, (b) 2, (c) 3, and (d) 6 layers. '1L', '2L', '3L' and '6L' represent 1, 2, 3 and 6 layers, respectively. 'GN' and 'GN-Circle' represent pristine graphene and graphene with circular nanopores, respectively.

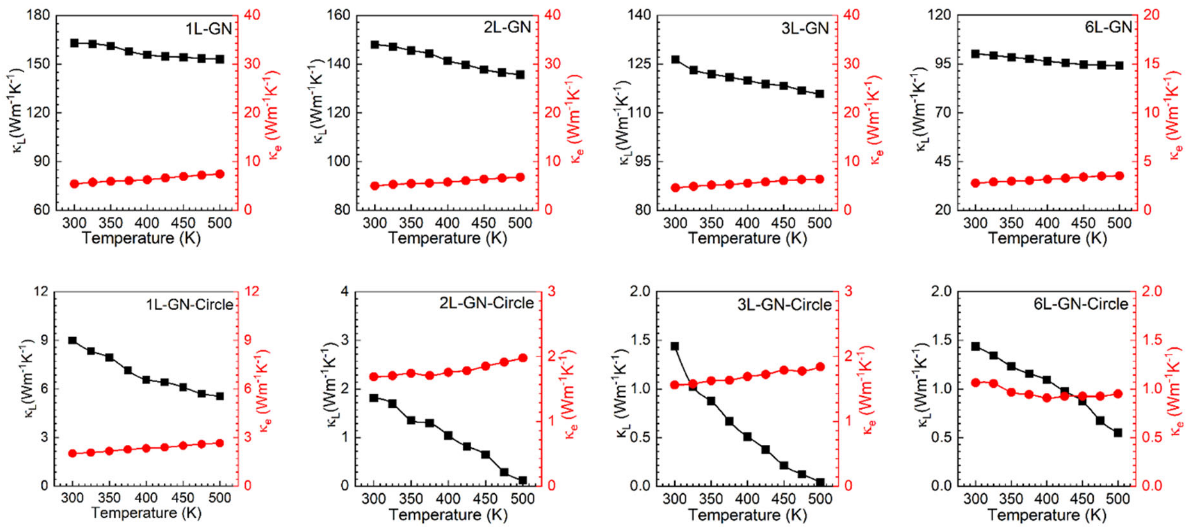

Supplementary Fig. 13 - Temperature-dependent electron thermal conductivity (κ_e) and lattice thermal conductivity (κ_L) of graphene with circle nanopore and different number of layers. The presence of nanopores significantly decreases κ_L. '1L', '2L', '3L' and '6L' represent 1, 2, 3 and 6 layers, respectively. 'GN' and 'GN-Circle' represent pristine graphene and graphene with circular nanopores, respectively.

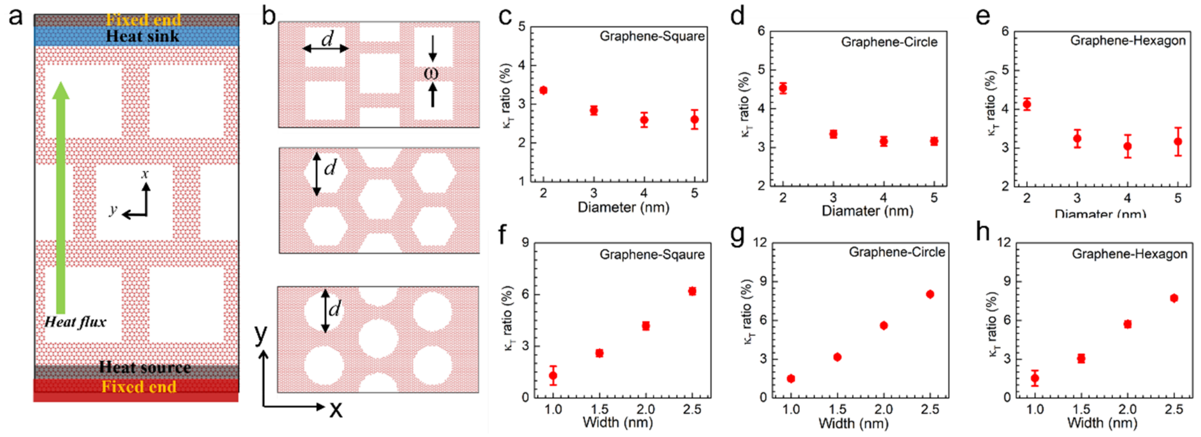

Supplementary Fig. 14 - Molecular dynamics simulation of the effect of pore structure on thermal conductivity. (a) Molecular model for the simulation of the thermal conductivity, κ_T, of porous monolayer graphene. (b) Atomic configurations of nanoporous monolayer graphene. κ_T of ordered porous graphene with different (c-e) pore sizes (with a fixed nanoribbon width, ω, of 1.5 nm) and (f-h) nanoribbon widths between adjacent nanopores (with a fixed nanopore size, d, of 4 nm). Error bars represent standard deviations from three independent measurements.

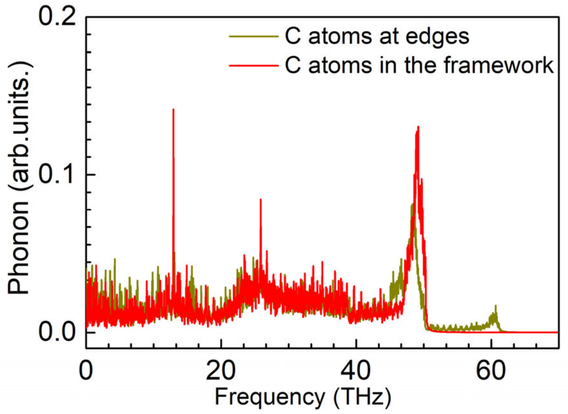

Supplementary Fig. 15 - Phonon intensity of carbon atoms in graphene nanoribbons with zigzag-shaped edges. The phonon intensity of carbon atoms at the pore edges (yellow curve) and in the framework of the graphene nanoribbon (away from the edges; red curve). The nanoribbon width is 5 nm.

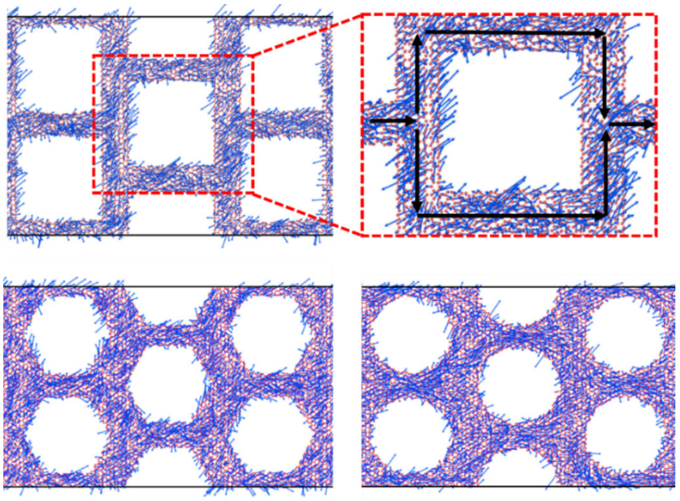

Supplementary Fig. 16 - Spatial distribution of the heat flux of porous graphene nanosheets. Vector arrows indicate the heat flux on each atom of porous graphene. The black arrows in the enlarged image of the pore show the global heat flux transport pathways.

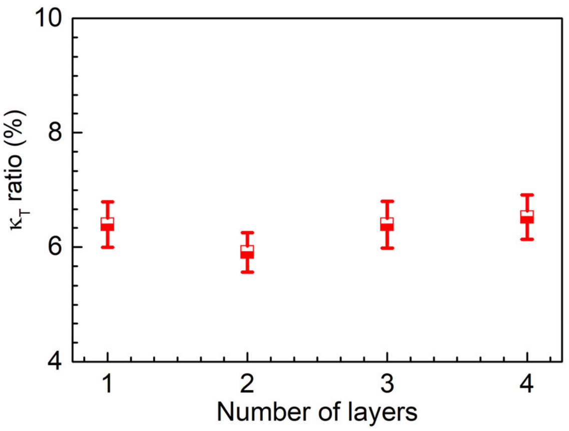

Supplementary Fig. 17 - Ratio of the thermal conductivity, κ_T, of nanoporous graphene over nonporous graphene with the same number of layers. The pore size, *d*, was set at 4 nm and the nanoribbon width, *ω*, was set at 2.5 nm. Error bars represent standard deviations from three independent measurements.

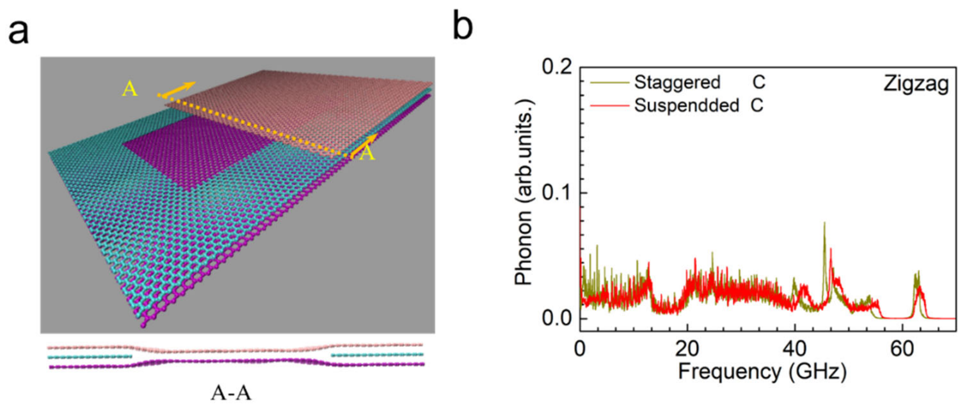

Supplementary Fig. 18 - Molecular dynamics simulation of the effect of the overlap ratio of pores on the thermal conductivity of graphene. (a) Scheme of a trilayer graphene structure with a pore in the middle graphene layer. (b) Phonon density of states of carbon atoms in a 3-layer graphene structure with an overlap ratio of 0 (no overlapping; red curve) and 1 (complete overlapping of the pores by the other graphene layers; yellow curve).

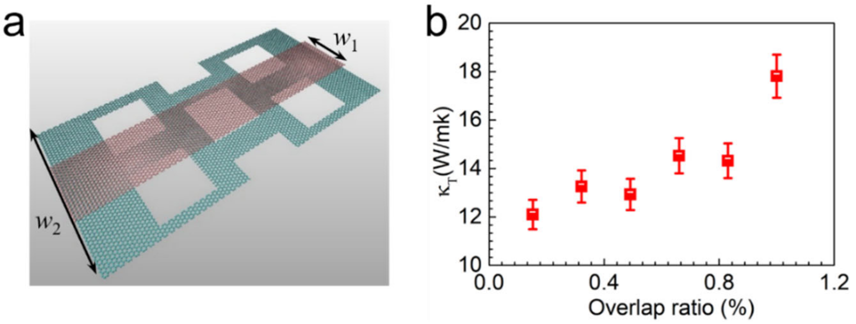

Supplementary Fig. 19 - The effect of overlap ratio on the thermal conductivity of ordered porous graphene. (a) Atomic configuration of a graphene layer with nanopores covered by a defect-free graphene nanoribbon. (b) Thermal conductivity, κ_T, of a nanoporous nanoribbon as a function of overlap ratio defined as w_1/w_2. Error bars represent standard deviations from three independent measurements.

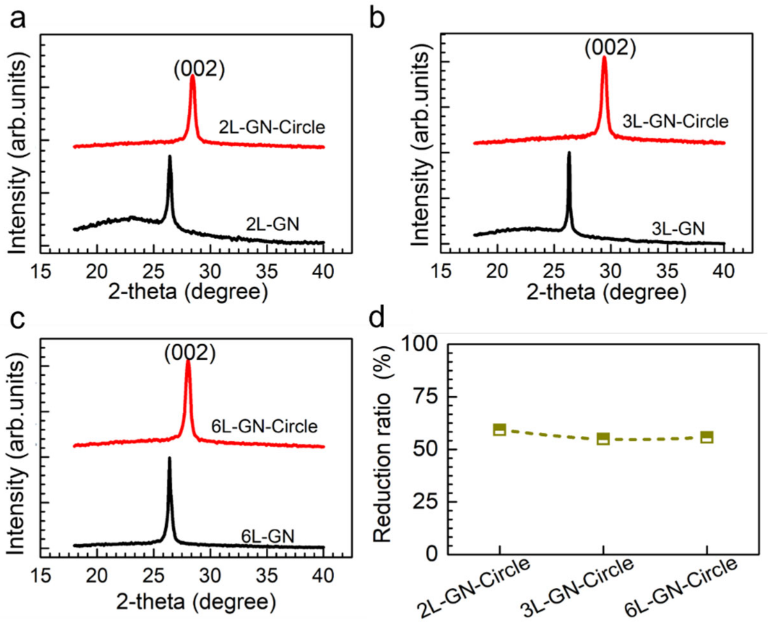

Supplementary Fig. 20 - Effect of the pore structure on the interlayer spacing of graphene. XRD (002) pattern of porous and nonporous graphene with (a) 2, (b) 3, and (c) 6 layers. (d) The reduction ratio of the interlayer spacing of porous graphene over nonporous graphene with the same graphene layer number. Based on the diffraction angle, the spacing distance was calculated based on Bragg's law. Error bars represent standard deviations from three independent measurements. '2L', '3L' and '6L' represent 2, 3 and 6 layers, respectively. 'GN' and 'GN-Circle' represent pristine graphene and graphene with circular nanopores, respectively.

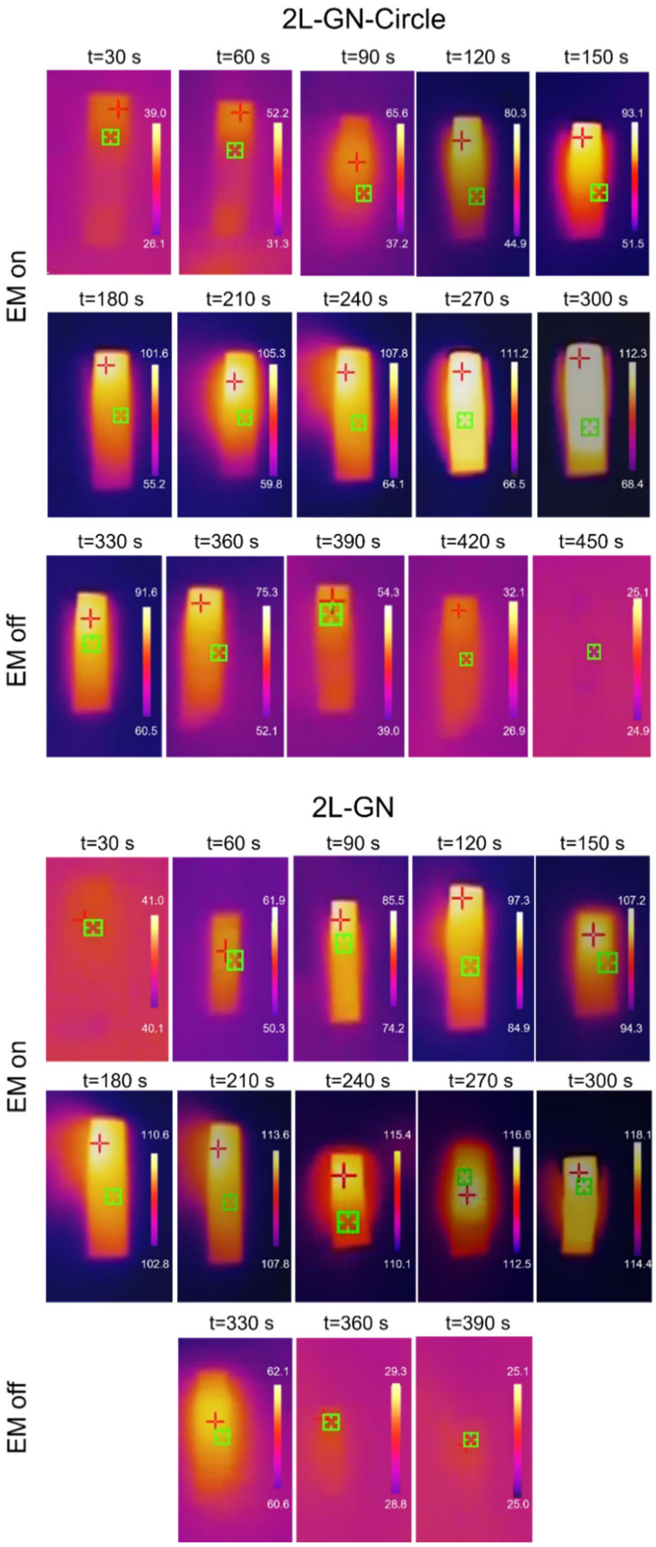

Supplementary Fig. 21 - Spatial and temporal temperature profile of nonporous graphene and ordered porous graphene upon EM wave irradiation using IR camera. The graphene strip width is 5 mm. '2L-GN' and '2L-GN-Circle' represent bilayer graphene without and with circular nanopores, respectively.

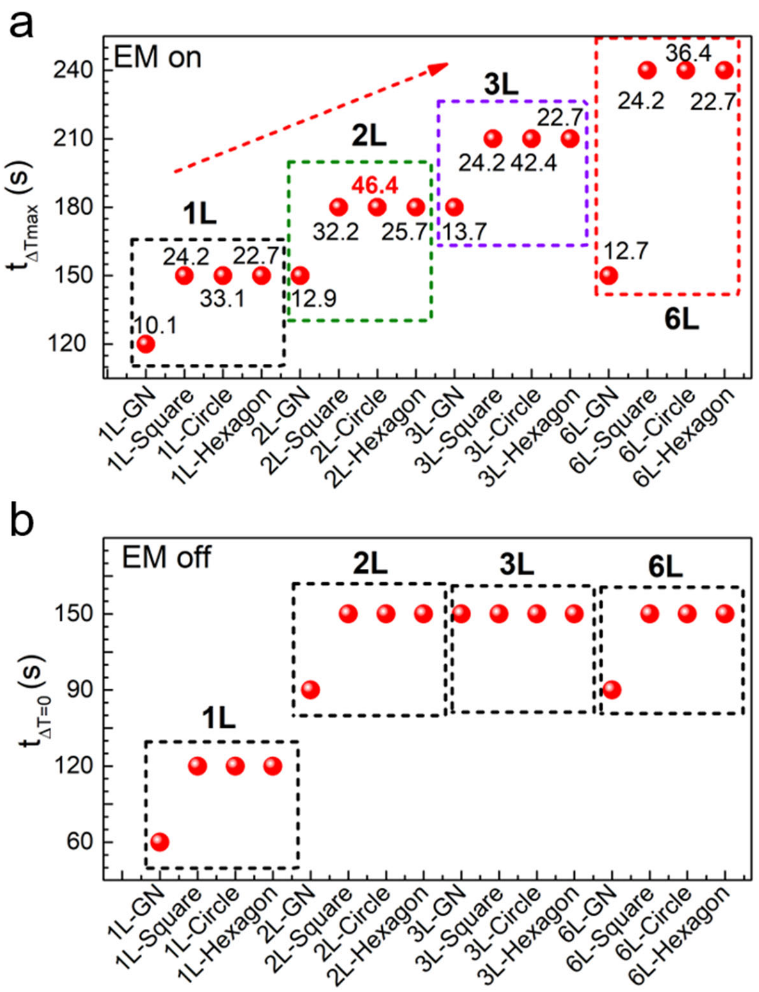

Supplementary Fig. 22 - Effect of pore structure on the temperature evolution dynamics in graphene. (a) Time required for graphene film to achieve a maximum temperature gradient upon EM radiation. The number labeled next to each data point represents the maximum temperature gradient in Kelvin. (b) Time required for graphene film to relax to a uniform temperature profile (no temperature gradient) after the removal of EM radiation. '1L', '2L', '3L' and '6L' represent 1, 2, 3 and 6 layers, respectively. 'GN' and 'GN-Circle' represent pristine graphene and graphene with circular nanopores, respectively.

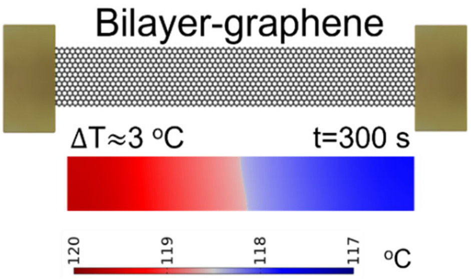

Supplementary Fig. 23 - Finite-element simulation of the temperature profile of nonporous bilayer graphene upon EM radiation. Δ*T* represents the temperature difference of the bilayer graphene.

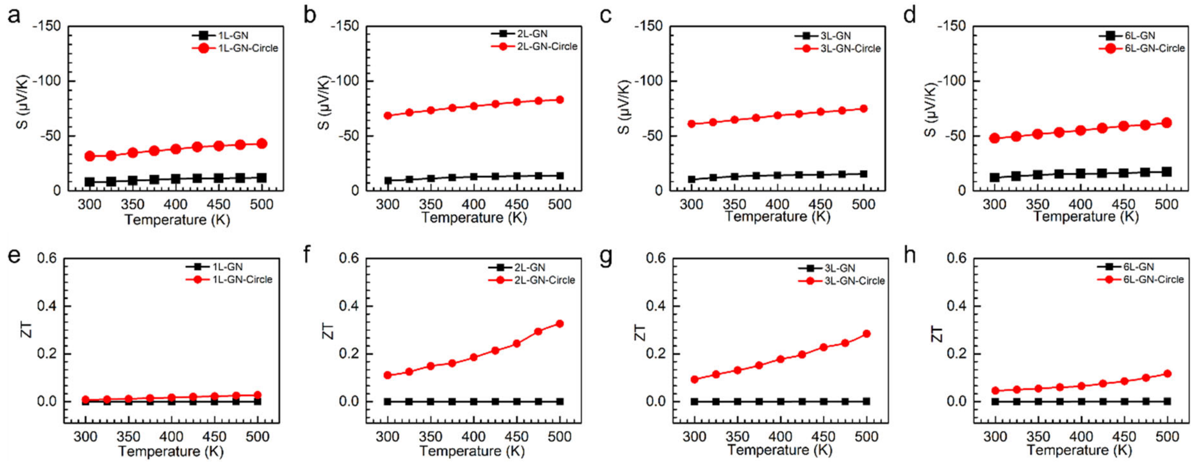

Supplementary Fig. 24 - Temperature-dependent Seebeck coefficient (*S*) and *ZT* value of graphene with circle nanopore and different numbers of layers. (a-d) *S* and (e-h) *ZT* value of porous graphene with (a, e) 1, (b, f) 2, (c, g) 3, and (d, h) 6 layers as a function of temperature. '1L', '2L', '3L' and '6L' represent 1, 2, 3 and 6 layers, respectively. 'GN' and 'GN-Circle' represent pristine graphene and graphene with circular nanopores, respectively.

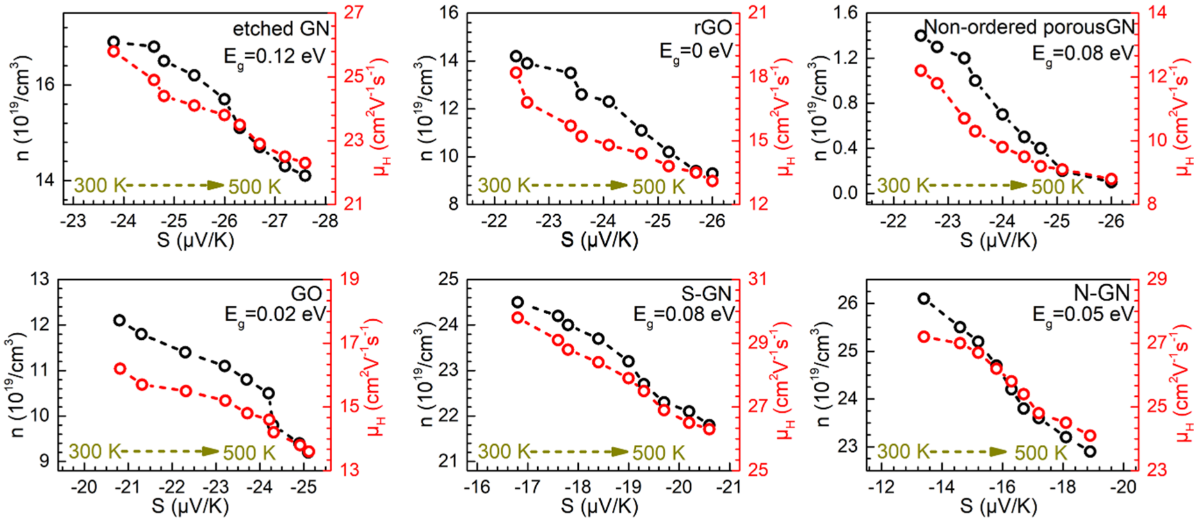

Supplementary Fig. 25 - Hall carrier density (*n*) and mobility (μ_H) of different graphene structures as a function of the Seebeck coefficient (*S*). The graphene structures include etched graphene, reduced graphene oxide, polydisperse porous graphene, graphene oxide, and S or N-doped graphene. The absolute Seebeck coefficients of the above graphene structures are measured to be less than 30 μV/K. 'GN', 'rGO', 'GO', 'S-GN' and 'N-GN' represent graphene, reduced graphene oxide, graphene oxide, sulfur-doped graphene and nitrogen-doped graphene, respectively.

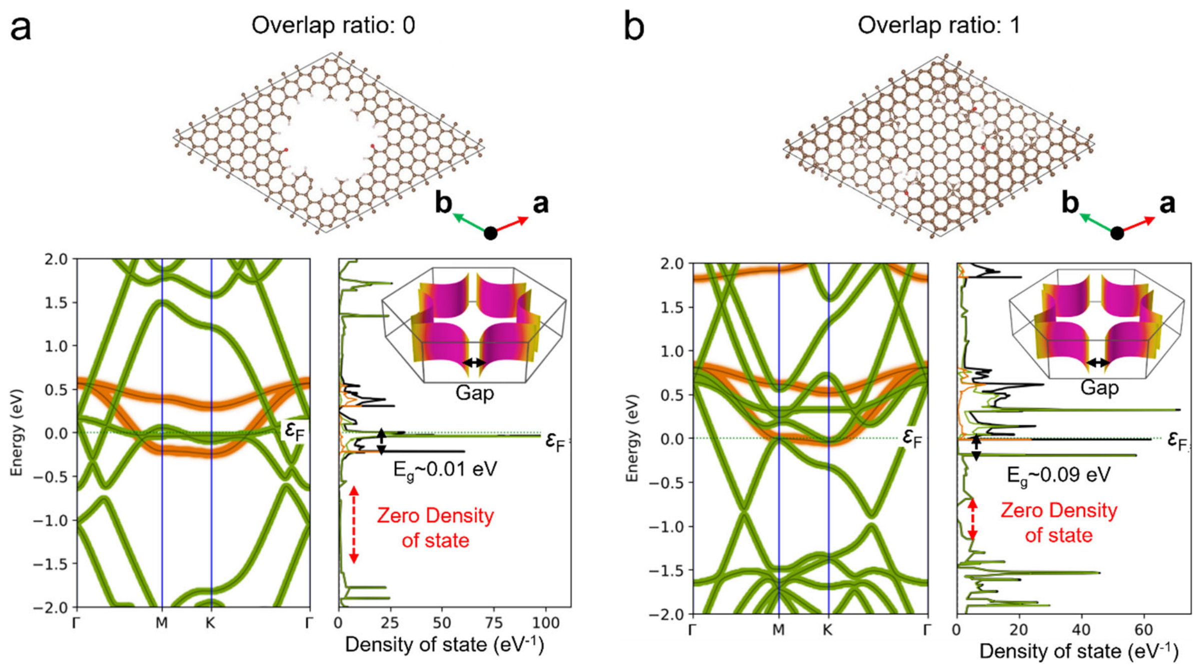

Supplementary Fig. 26 - Density functional theory (DFT) calculations of the band structures and projected density of state of porous bilayer graphene with different overlap ratios. The overlap ratio of porous bilayer graphene is (a) 0 and (b) 1. The bandgap (E_g) and zero density of state are indicated by the black and red double-headed arrows, respectively. The diameter of the nanopores is 1.2 nm. Insets show the calculated Fermi surface of bilayer nanoporous graphene with different overlap ratios. Compared to nonporous bilayer graphene, the Fermi surface of nanoporous bilayer graphene with different overlap ratios exhibits a remarkable splitting behavior. ε_F represents the Fermi level.

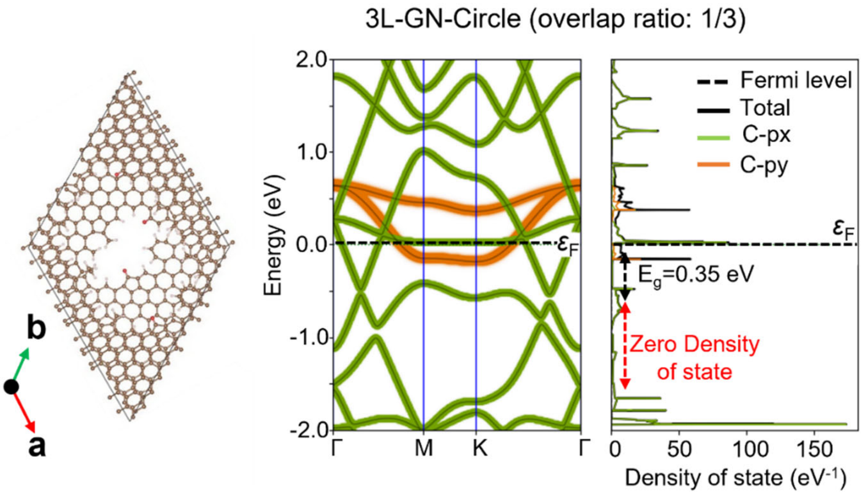

Supplementary Fig. 27 - DFT calculations of the band structures and projected density of state of trilayer graphene with circular pore structure. The overlap ratio is 1/3. The bandgap (E_g) and zero density of state are indicated by the black and red double-headed arrows, respectively. The diameter of the nanopores is 1.2 nm. '3L-GN-Circle' represents the trilayer graphene with circular nanopores. ε_F represents the Fermi level.

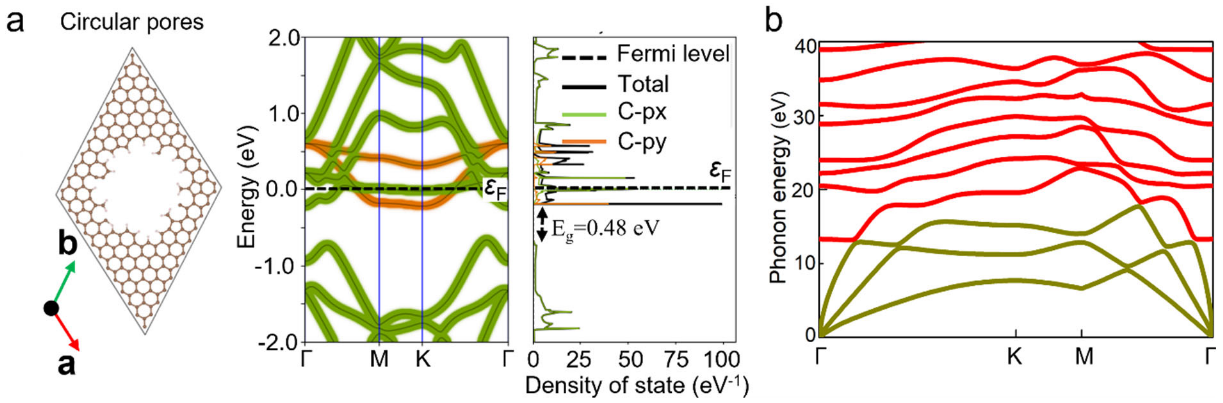

Supplementary Fig. 28 - DFT calculation of band structure, projected electron density of state, and phonon density of state of porous monolayer graphene. (a) Projected electron density of state and (b) phonon density of state of porous monolayer graphene. Black double-headed arrows indicate bandgap (E_g). The diameter of the nanopores is 1.2 nm. ε_F represents the Fermi level.

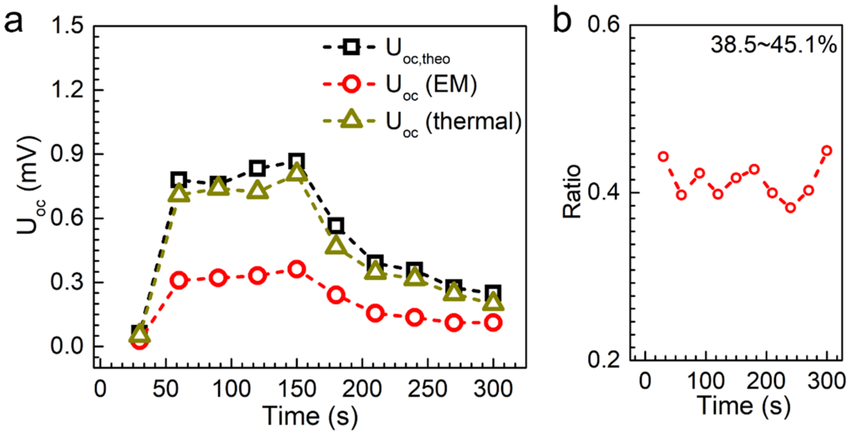

Supplementary Fig. 29 - Open-circuit voltage of bilayer nonporous graphene-based device. (a) Experimental and theoretical open-circuit voltage [$U_{oc\ (EM)}$ and $U_{oc,theo}$; respectively] of pristine graphene under EM radiation. In the yellow curve, the temperature gradient is applied by using an external thermal source instead of EM radiation and corresponding experimental open-circuit voltage [$U_{oc\ (thermal)}$]. (b) The ratio of U_{oc} of nonporous graphene under an EM field over the theoretical U_{oc}.

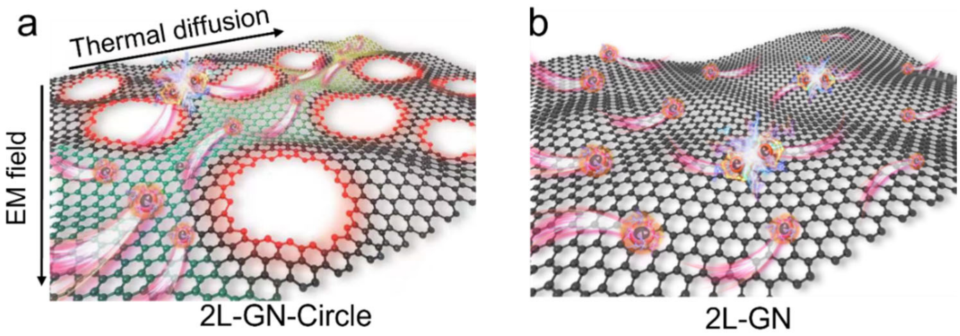

Supplementary Fig. 30 - Schematic illustration of transport of electrons in a graphene layer. Electron transport in graphene (a) with and (b) without circular pores. The presence of nanopores will confine/guide the transport of electrons in graphene nanoribbons between the pores. '2L-GN' and '2L-GN-Circle' represent bilayer graphene without and with circular nanopores, respectively.

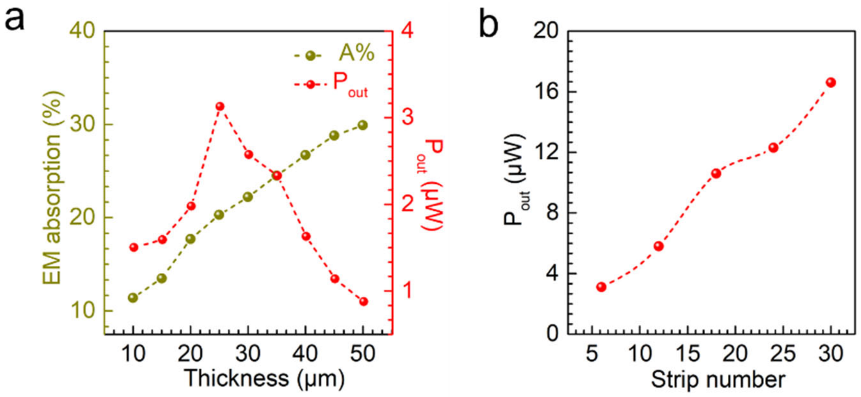

Supplementary Fig. 31 – Effect of device dimension on the output power. (a) EM absorption and output power (P_{out}) of the ordered nanoporous graphene-based device as a function of the thickness of the ordered nanoporous graphene strips. The device consisted of six ordered nanoporous graphene strips with each strip being 25 mm long and 5 mm wide. The thickness was varied from 10 to 25 μm. (b) P_{out} of the ordered nanoporous graphene-based device as a function of the number of strips. Each strip was 25 mm long, 5 mm wide, and 25 μm thick.

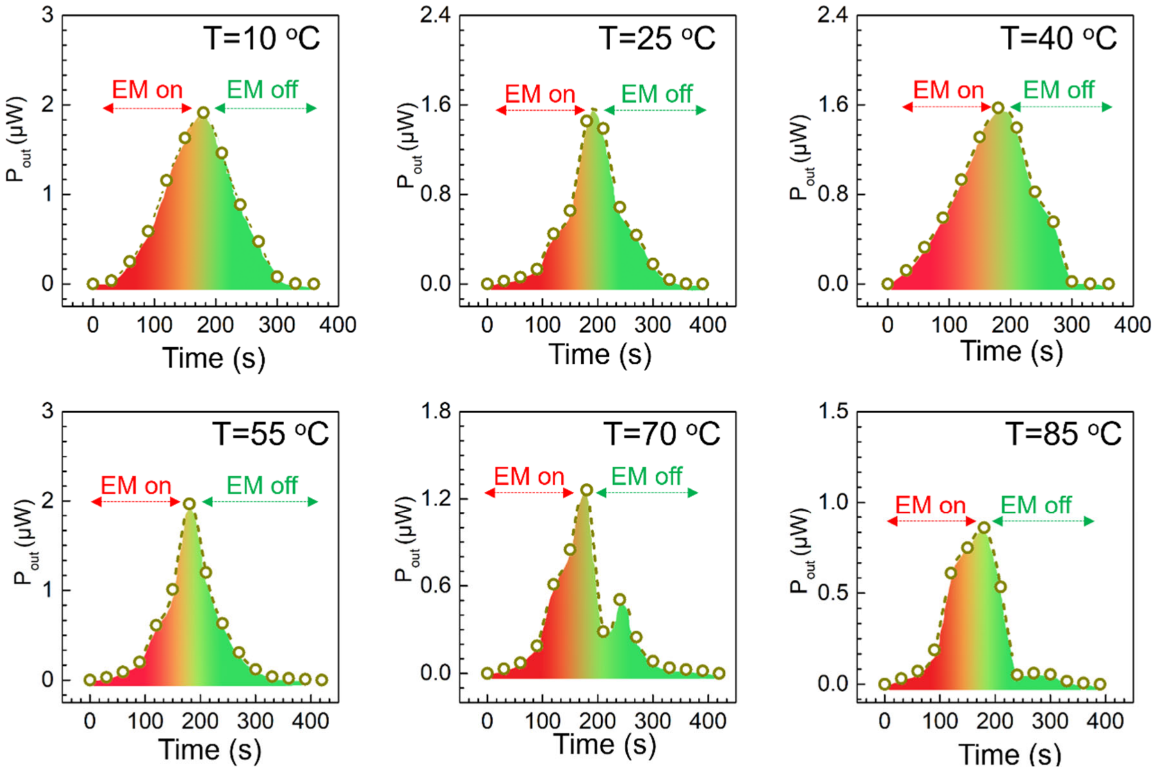

Supplementary Fig. 32 - Output power (P_{out}) of a bilayer porous graphene-based device as a function of EM radiation at different temperatures. The red and green double-headed arrows represent the time when EM radiation is on and off, respectively.

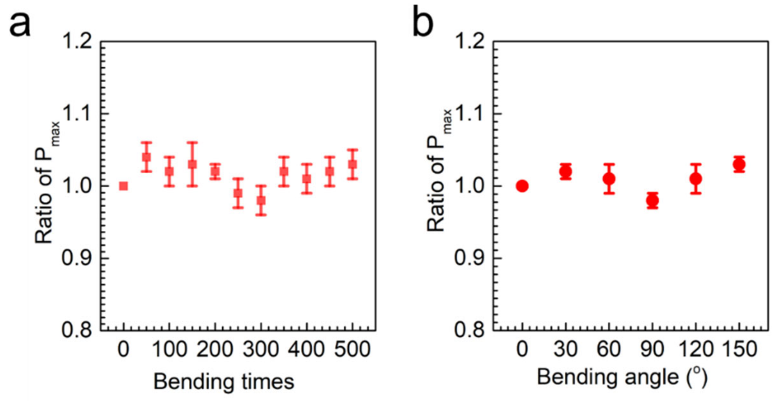

Supplementary Fig. 33 - Output power by a flexible ordered porous graphene device during bending. Maximum output power (P_{max}) as a function of (a) the number of times and (b) the angle of bending of the ordered porous graphene-based flexible device. Error bars represent standard deviations from three independent measurements.

Supplementary Tables

Supplementary Table 1. Representative EM frequency ranges for 3G, 4G, and 5G wireless technology.

Band	3G (GHz)	4G (GHz)	5G (GHz)
1	1.71-1.78	1.88-1.90	3.30-3.40
2	1.88-1.90	2.32-2.37	3.40-3.60
3	2.01-2.02	2.57-2.63	4.80-5.00

Supplementary Table 2. Permittivity, polarization frequency, and EM dissipation factor of conventional EM dissipation materials.

Material	ε'	ε''	Polarization frequency (GHz)	EM dissipation factor, η	Ref.
Fe_3O_4/Fe	19.3-14.2	3.9-2.8	8.4, 13.5	~ 4.0	33
Fe_3O_4/Cu	10.7-6.1	2.6-1.0	10.8	~ 3.0	34
$FeSn_2$/Sn	9.3-3.1	4.7-1.4	8.2	~ 4.8	35
Fe@Ag	6.1-4.3	0.7-0.1	5.1, 6.0, 11.7	< 0.1	36
FeSiAl/NiZn	9.1-7.5	1.4-0.1	10.2	< 1.5	37
Co@CoO	9.2-8.6	1.6-0.1	3.9, 9.3	< 1.7	38
CoO/Co_3O_4	7.2-4.9	2.0-1.2	8.6, 12.6	< 2.2	39
Co-Ni-P	15.4-8.0	8.0-0.3	6.0, 8.1, 12.6	~ 8.1	40
Ni@Ni_2O_3	14.1-12.1	1.2-0.2	15.9	< 1.3	41
Ni@SnO_2	8.2-7.7	4.8-1.9	9.3, 14.2	< 5.0	42
Cu@Ni	23.1-8.7	14.9-0.9	7.5, 10.5, 17.2	< 15	43
$SrZnCoFe_{16}O_{27}$	6.1-6.0	0.3-0.2	10.4	< 0.4	44
TiN/Ni/C	17.3-11.8	6.1-4.2	9.4, 12.1	< 6.5	45
MnO_2/NPC	13.2-8.5	4.0-3.5	10.4, 12.8, 15.4	< 4.2	46
C/MnO/Co	10.3-4.8	6.5-2.2	8.9, 13.6, 16.0	< 6.7	47
Fe/NPC	13.2-6.2	6.2-4.1	9.5	< 6.5	48
Fe/CNT	15.6-7.2	5.9-4.1	16.0	< 6.0	49
($Fe_{70}Ni_{30}$)/C	12.1-7.3	6.0-3.2	9.2, 14.8	< 6.2	50
Fe-Co/NC/rGO	8.0-2.5	5.3-1.4	9.8, 13.2	< 5.5	51
Fe@C@$BaTiO_3$	16.3-9.4	5.1-3.8	10.8, 14.8	< 5.5	52
Fe_2O_3/C/rGO	7.8-4.8	1.3-0.1	16.6	< 1.5	53
Fe_3O_4/Fe@C@MoS_2	10.6-7.7	2.4-1.5	11.5, 16.9	< 2.5	54
Co/C	6.7-5.0	2.2-0.9	13.1	< 2.3	55
Co/CNT	18.4-3.2	20.7-1.3	7.5	< 21.0	56
CoAl@C	20.0-10.0	8.3-2.1	12.1	< 8.5	57
CoMo@HNCP	7.9-4.2	4.5-1.1	8.2, 11.9, 15.8	< 4.7	58
Co@SiO_xC	5.6-5.0	1.9-0.5	11.7, 14.0, 15.5	< 2.1	59
Co_3O_4/rGO	12.2-8.0	6.5-2.5	12.1, 14.9	< 6.8	60
Ni/CNTs	6.4-1.8	6.8-3.7	8.5	< 7.0	61
Ni/TiO_2/C	13.0-8.9	9.2-0.1	15.4, 16.5, 17.7	< 9.4	62
Ni@C@ZnO	11.8-8.7	5.7-3.6	7.6, 12.0	< 5.8	63

$NiO@SiO_2@GN$	6.1-3.8	2.9-1.0	3.9, 15.6	< 3.0	64
$Ni@Co_3O_4$/rGO	9.8-5.2	4.0-3.3	9.7, 15.6, 16.9	~ 4.1	65
Cu/C	21.7-20.5	13.9-5.3	15.3	~ 14.1	66
$Sn@Mo_2C$/C	13.0-6.5	5.5-3.3	11.7, 13.1, 14.4	~ 5.6	67
$CuFe_2O_4/MoS_2$	8.5-6.0	2.4-2.3	N.A.	< 2.5	68
$MnFe_2O_4$/MgO	10.0-7.0	4.0-2.0	10.0	< 4.5	69
Co@C	16.0-6.0	28.0-8.0	13.7	< 28.0	70
$CoFe_2O_4$/porous C	20.0-10.0	9.5-4.5	15.1	< 9.5	71
Fe@Ag	7.0-6.0	< 0.1	10.0	< 0.2	72

Supplementary Table 3. Thermal conductivity of a variety of modified graphene, 2D materials, and porous carbons.

Material	Thermal conductivity (W/m·K)	Temperature (K)	Ref.
Isotopically-modified graphene	> 4,000	320	73
Graphene laminate	40-90	323	74
Reduced graphene oxide	~ 6.5	500	75
CVD-synthesized graphene	28	350	76
Br-doped graphene	~ 78	350	77
Graphene quantum dots/CNT	58.9	298	78
Single CNT	2,000	320	79
Monolayer MoS_2	87.8	N.A.	80
$Ti_3C_2T_x$	108	600	81
WSe_2	~ 10	800	82
Porous SiC ceramic	~ 1.5	1,000	83
Porous carbon	4.5	N.A.	84
Few-layer WS_2	32	298	85
Bilayer WS_2	32	298	86
Al-CNT	~ 80	573	87

Supplementary Table 4. Dependence of $d_{1\text{-}4}$ and thermal conductivity of a four-layer graphene nanosheet on its shifting distance, $S_{2\text{-}4}$.

$S_{2\text{-}4}$ (Å)	$d_{1\text{-}4}$ (Å)	Thermal conductivity (W/mK)
0	10.50	13.80
20	9.53	13.26
40	9.52	13.06
60	10.42	13.49
80	9.46	12.68
100	9.52	13.07

Supplementary Table 5. Absolute Seebeck coefficient of conventional 2D nanomaterials.

Material	Absolute Seebeck coefficient (μV/K)	Temperature (K)	Ref.
Twisted bilayer graphene	~ 11	300	88
Multilayer graphene	~ 20	< 300	89
S-doped graphene	~ 30	300	90
Graphene nanoribbon	~ 30	300	91
SnS_2	~ 140	300	92
Bi-doped SnSe	~ 100	300	93
$Mo_2TiC_2T_x$	~ 50	800	94
p-typed MoS_2	~ 50	450	95
2H-MoS_2	~ 30	323	96
Graphene/PVP	45.1	500	97
Si doped Graphene	~ 100	300	98
Graphene/PEDOT:PSS	32.3	100	99
Graphene/MoS_2	50	550	100
Graphene/C_{60}	55	N.A.	101
Graphene/BiSrTe alloy	~ 165	300	102
Graphene/carbon aerogel	~ 15	298	103
CNT/Cu_2Se	~ 100	450	104
CF/Bi_2Te_3	~ 50	300	105
MXene-PEDOT:PSS	57.3	400	106
SnTe/WSe_2	~ 50	600	107

Supplementary Table 6. ZT values of convertional thermoelectric materials and graphene

Sample	Maximum *ZT* (Temperature range)	*ZT* at 500K	Ref.
Single layer graphene	<0.01 at 600K (300K-600K)	<0.01	108
Defective graphene	<0.01 at 300K (300K)	N.A.	109
Graphene/Bi_2Te_3	0.55 at 500K (300K-500K)	0.55	110
Graphene/$CoSb_3$	0.61 at 800K (300K-800K)	~0.15	111
Graphene/$Bi_{2-x}Te_3$	0.2 at 480K (300K-480K)	N.A.	112
Graphene/$CuInTe_2$	0.4 at 700K (300K-700K)	0.1	113
Cu_2Se	1.2 at 1000K (300K-1000K)	~0.1	114
$AgMnSbTe_3$	1.4 at 800K (300K-800K)	~0.6	115
Ga-doped PbTe	1.55 at 723K (300K-723K)	~0.5	116
$AgMnGeSbTe_4$	1.05 at 773K (300K-773K)	~0.6	117
$Pb_7Bi_4Se_{13}$	1.35 at 800K (300K-800K)	~0.5	118
(Pb, Ge, Sb, Cd)-doped SnTe	1.5 at 800K (300K-800K)	~0.6	119
$Cu_{12}Sb_4S_{13}$-based alloy	1.15 at 723K (300K-723K)	0.4	120
Cu_2SnSe_3	1.6 at 823K (300K-823K)	<0.2	121
$Bi_2Si_2Te_6$	0.51 at 623K (300K-623K)	<0.3	122
$ZrRu_{1+x}Sb$	0.2 at 973K (300K-973K)	<0.1	123
2L-GN-Circle	0.33 at 500K (300K-500K)	0.33	This work

Supplementary Table 7. Maximum power density and temperature difference of current electricity generating materials that utilize temperature gradients.

Material	$P_{density}$ (W/m^3)	ΔT (K)	$P_{density}/\Delta T$ (W/m^3·K)	Ref.
n/*p*-SWCNT	4.16	30	0.138	124
TENG	~ 1.4	80	1.75×10^{-2}	125
n-type Ag_2Se	23	30	7.66×10^{-1}	126
PEDOT:PSS/Cu_2Se	91	30	3.0	127
TE/PEDOT:PSS	572	40	14.3	128
PANI/CNT/Te	62.4	40	1.56	129
PEDOT:PSS/TiS_2[(HA)(NMF)]	80.0	40	2.0	130
PEDOT:PSS/Te/Cu_7Te_4	39.5	39.1	1.01	131
PVP/Ag/Ag_2Te	34.1	39.6	8.6×10^{-1}	132
Ag_2Se/Ag/PEDOT	74.7	27	2.76	133
CNT/PEG	12.5	25	0.5	134
$NbSe_2/WS_2$	7.3×10^{-1}	60	1.21×10^{-3}	135
$Bi_{0.5}Sb_{0.15}Te_3$	22.8	N.A.	N.A.	136
$(Nb_{0.8}Ta_{0.2})_{0.8}Ti_{0.2}FeSb$	211	655	0.32	137
Ag-Se-TENG	32.1	110	0.29	138
Bi_2Te_3-TENG	92.0	52.5	1.75	139
$Ce_yFe_3CoSb_{12}$	21.0	570	3×10^{-2}	140
Bi_2Te_3	11.4	N.A.	N.A.	141
$Ge_{0.84}Pb_{0.1}Sb_{0.06}$	176.0	425	0.41	142
Se-Co-Ni alloy	43.6	500	0.87	143

Supplementary Table 8. Thermoelectric coefficients of state-of-the-art thermoelectric materials and bilayer graphene with circular nanopores.

p-type	*n*-type	Dimension (mm)	*N*	ΔT<br>T_c-T_h (K)	Device C_{TE}*<br>(single strip)	Ref.
$Yb_{0.3}Co_4Sb_{12}$	$Ce_{0.85}Fe_3CoSb_{12}$	20×20×14.5	16	574<br>313-887	10.2%<br>(0.64%)	144
Graphene modifed-$Yb_yCo_4Sb_{12}$	$Ce_yFe_3CoSb_{12}$	4×4×12	16	557<br>300-857	24%<br>(1.5%)	145
$Mg_{3.1}Co_{0.1}Sb_{1.5}Bi_{0.49}Te_{0.01}$	$ZrCoBi_{0.65}Sb_{0.15}Sn_{0.2}$	3.6×3.6×4.4	4	400<br>373-773	10.6%<br>(2.7%)	146
$Mg_{0.99}Cu_{0.01}Ag_{0.97}Sb_{0.99}$	$Mg_{3.2}Sb_{1.5}Bi_{0.49}Te_{0.01}Cu_{0.01}$	3×2×2	8	320<br>279-593	7.3%<br>(0.91%)	147
Ni-doped MgAgSb	Bi_2Te_3	N.A.	2	225<br>293-568	10.0%<br>(5.0%)	148
Cu_2Se	$Ni/Ti/Yb/Co_4Sb_{12}$	10×4×4	16	680<br>293-973	9.1%<br>(0.57%)	149
ZrCoBi-based half-Heuslers	N.A.	4.6×1.5×2.4	2	500<br>323-823	9.0%<br>(4.5%)	150
TaFeSb-based half-Heuslers	N.A.	11×2.6×2.7	2	656<br>317-973	11.4%<br>(5.7%)	151
$(Bi, Sb)_2Te_3$	N.A.	N.A.	N.A.	200<br>298-758	9.0%<br>(0.05%)	152
$Yb_{0.09}Ba_{0.05}La_{0.05}Co_4Sb_{12}$	$Mm_{0.3}Fe_{1.46}Co_{2.54}Sb_{12.05}$	4×4×4	64	460<br>298-758	7.0%<br>(0.11%)	153
N.A.	Reduced graphene oxide	25×5×0.01	6	200<br>300-500	<0.1%<br>(<0.01%)	This work
N.A.	Graphene prepared by CVD	25×5×0.01	6	200<br>300-500	<0.1%<br>(<0.01%)	This work
N.A.	2L-GN-Circle	25×5×0.01	6	200<br>300-500	14.4%<br>(2.4%)	This work

*Thermoelectric coefficient C_{TE} is calculated by $C_{TE} = \frac{T_h - T_c}{T_h} \frac{\sqrt{1+\overline{ZT}}-1}{\sqrt{1+\overline{ZT}}+T_c/T_h}$, where T_h and T_c are the temperatures of the hot and cold ends ($\Delta T = T_h - T_c$), respectively[154]. $\overline{ZT}$ is the average ZT over the temperature range, which is calculated by the integration method (i.e., a fourth-order polynomial fitting of the ZT curve).

Supplementary Table 9. Experimental parameters for the synthesis of ordered porous graphene with different nanopore shapes.

Fe_3O_4 nanoparticle shape	Diameter (nm)	$V_{Oleic\ acid}$ (mL)	Heat rate (ºC/min)
Cubic	7±1	10	8±1
Spherical	6±1	12	8±1
Hexagonal	16±2	9	8±1

Supplementary References

1. Bagchi, B. *et al.* Polarization relaxation, dielectric dispersion, and solvation dynamics in dense dipolar liquid. *J. Chem. Phys.* **90**, 7338 (1989).
2. Dou, Y. K. *et al.* The enhanced polarization relaxation and excellent high-temperature dielectric properties of N-doped SiC. *Appl. Phys. Lett.* **104**, 052102 (2014).
3. Zeng, X. J. *et al.* Electromagnetic microwave absorption theory and recent achievements in microwave absorbers. *Carbon* **168**, 606-623 (2020).
4. Qin, F. *et al.* A review and analysis of microwave absorption in polymer composites filled with carbonaceous particles. *J. Appl. Phys.* **111**, 061301 (2012).
5. Wang, H. *et al.* Optimal electromagnetic-wave absorption by enhanced dipole polarization in Ni/C nanocapsules. *Appl. Phys. Lett.* **101**, 083116 (2012).
6. Lv, H. *et al.* Engineering defects in 2D g-C_3N_4 for wideband, efficient electromagnetic absorption at elevated temperature. *J. Mater. Chem. A* **9**, 19710-19718 (2021).
7. Lv, H. *et al.* Doping strategy to boost the electromagnetic wave attenuation ability of hollow carbon spheres at elevated temperatures. *ACS Sustainable Chem. Eng.* **6**, 1539-1544 (2018).
8. Lou, Z. C. *et al.* An angle-insensitive electromagnetic absorber enabling a wideband absorption. *J. Mater. Sci. Technol.* **113**, 33 (2022).
9. Ao, D.W. *et al.* Novel thermal diffusion temperature engineering leading to high thermoelectric performance in Bi_2Te_3-based flexible thin-films. *Adv. Sci.* **5**, 2103547 (2022).
10. Chang, C. W. *et al*. Breakdown of Fourier's law in nanotube thermal conductors. *Phys. Rev. Lett.* **101**, 075903 (2008).
11. Feng, T. L. *et al.* Ultra-low thermal conductivity in graphene nanomesh. *Carbon* **101**, 107 (2016).
12. Wei, C. *et al.* Thermal conductivity of graphene kirigami: ultralow and strain robustness. *Carbon* **104**, 203 (2016).
13. Wang, Y. *et al.* Edge effect on thermal transport in graphene nanoribbons: A phonon localization mechanism beyond edge roughness scattering. *Appl. Phys. Lett.* **101**, 013101 (2012).
14. Guo, Z. X. *et al.* Thermal conductivity of graphene nanoribbons. *Appl. Phys. Lett.* **95**, 163103 (2009).
15. Evans, W. J. et al. Thermal conductivity of graphene ribbons from equilibrium molecular dynamics: effect of ribbon width, edge roughness, and hydrogen termination. *Appl. Phys. Lett.* **96**, 203112 (2010).
16. Wei, Z. Y. *et al.* In-plane lattice thermal conductivities of multilayer graphene films. Carbon **49**, 2653 (2011).
17. Cao, H. Y. *et al.* Layer and size dependence of thermal conductivity in multilayer

graphene nanoribbons. *Phys. Lett. A* **376**, 525 (2012).

18. Gho, S. *et al.* Dimensional crossover of thermal transport in few-layer graphene. *Nat. Mater.* **9**, 555 (2010).
19. Li, X. B. *et al.* Strain effects on the thermal conductivity of nanostructures. *Phys. Rev. B* **81**, 245318 (2010).
20. Zhu, H. T. *et al.* Discovery of ZrCoBi based half-heuslers with high thermoelectric conversion efficiency. *Nat. Commun.* **9**, 2497 (2018).
21. Zhu, H. T. *et al.* Discovery of TaFeSb-based half-heuslers with high thermoelectric performance. *Nat. Commun.* **270**, 10 (2019).
22. Blackburn, J. L. *et al.* Carbon-nanotube-based thermoelectric materials and devices. *Adv. Mater.* **11**, 1704386 (2018).
23. Xu, S. D. *et al.* Conducting polymer-based flexible thermoelectric materials and devices: from mechanisms to applications. *Prog. Mater. Sci.* **121**, 100840 (2021).
24. Hafner, J. *et al.* Ab-initio simulations of materials using VASP: Density-functional theory and beyond. *J. Comput. Chem.* **29**, 2044 (2008).
25. Perdew, J. P. *et al.* Generalized gradient approximation made simple. *Phys. Rev. Lett.* **77**, 3865-3868 (1996).
26. Kresse, G. *et al.* From ultrasoft pseudopotentials to the projector augmented-wave method. *Phys. Rev. B.* **59**, 1758 (1999).
27. Becke, A. D. *et al.* Density-functional thermochemistry. IV. A new dynamical correlation functional and implications for exact-exchange mixing. *J. Chem Phys.* **104**, 1040 (1996).
28. Grimme, S. et al. A consistent and accurate ab initio parametrization of density functional dispersion correction (DFT-D) for the 94 elements H-Pu. *J Chem Phys* **132,** 154104 (2010).
29. Blochl, P. E. *et al.* Projector augmented-wave method. *Phys. Rev. B* **50**, 17953 (1994).
30. Togo, A. *et al*. Frist principles phonon calculations in materials sciences. *Scrip. Mater.* **108**, 1-5 (2015).
31. Toyoshima, M. *et al*. Comparison of microwave and light wave communication systems in space applications. *Opt. Eng.* 47, 015003 (2007).
32. Heremans J. P. *et al.* Enhancement of thermoelectric efficiency in PbTe by distortion of the electronic density of states. *Science* **321**, 554 (2008).
33. Li, X. *et al.* Fabrication of three-dimensional flower-like heterogeneous Fe_3O_4/Fe particles with tunable chemical composition and microwave absorption performance. *ACS Appl. Mater. Interfaces* **11**, 19267-19276 (2019).
34. Fang, G. *et al.* Enhanced microwave absorption performance of Fe_3O_4/Cu composites with coexistence of nanospheres and nanorods. *J. Alloys Compd.* **817**, 152764 (2020).
35. Zhang, X. *et al.* Multiple-phase carbon-coated $FeSn_2$/Sn nanocomposites for high-frequency microwave absorption. *Carbon* **96**, 972-979 (2016).
36. Yang, P. A. *et al.* Optimization of Fe@Ag core–shell nanowires with improved impedance

matching and microwave absorption properties. *Chem. Eng. J.* **430**, 132878 (2022).

37. Lei, C. *et al.* Tunable dielectric loss to enhance microwave absorption properties of flakey FeSiAl/ferrite composites. *J. Alloys Compd.* **822**, 153674 (2020).
38. Deng, J. *et al.* Enhancing the microwave absorption properties of amorphous CoO nanosheet-coated Co (hexagonal and cubic phases) through interfacial polarizations. *J. Colloid Interface Sci.* **509**, 406-413 (2018).
39. Xie, X. *et al.* Phase and morphology evolution of high dielectric CoO/Co_3O_4 particles with Co_3O_4 nanoneedles on surface for excellent microwave absorption application. *Chem. Eng. J.* **396**, 125205 (2020).
40. Liu, J. *et al.* Microwave absorption properties of rod-shaped Co–Ni–P shells prepared by metallizing Bacillus. *Appl. Surf. Sci.* **257**, 2383-2386 (2011).
41. Wang, B. *et al.* Synthesis and enhanced microwave absorption properties of Ni@ Ni_2O_3 core–shell particles. *J. Alloys Compd.* **567**, 21-25 (2013).
42. Zhao, B. *et al.* Yolk–shell Ni@SnO_2 composites with a designable interspace to improve the electromagnetic wave absorption properties. *ACS Appl. Mater. Interfaces* **8**, 28917-28925 (2016).
43. Zhou, Y. *et al.* Core–shell chain-like Cu@Ni composite with dual absorption peaks to improve microwave absorption properties. *Nano* **13**, 1850052 (2018).
44. Azizi, P. *et al.* Magnetic and microwave absorption properties of $SrZnCoFe_{16}O_{27}$ powders synthesized by solution combustion method. *J. Alloys Compd.* **739**, 211-217 (2018).
45. Zhang, Y. *et al.* TiN/Ni/C ternary composites with expanded heterogeneous interfaces for efficient microwave absorption. *Compos. Part B-Eng.* **193**, 108028 (2020).
46. Yang, H. *et al.* 1D-3D mixed-dimensional MnO_2@ nanoporous carbon composites derived from Mn-metal organic framework with full-band ultra-strong microwave absorption response. *Chem. Eng. J.* **417**, 128087 (2021).
47. Zheng, S. *et al.* Facile Preparation of C/MnO/Co nanocomposite fibers for high-performance microwave absorption. *Compos. Part A-Appl. S.* **23**, 106814 (2022).
48. Heng, L. *et al.* Fe/nanoporous carbon hybrid derived from metal–organic framework for highly effective microwave absorption. *Appl. Organomet. Chem.* **33**, e4991 (2019).
49. Qi, X. *et al.* Preparation, electromagnetic and enhanced microwave absorption properties of Fe nanoparticles encapsulated in carbon nanotubes. *Mater. Sci. Eng. B-Adv.* **198**, 108-112 (2015).
50. Xie, Z. *et al.* Magnetic and microwave-absorption properties of graphite-coated (Fe, Ni) nanocapsules. *J. Mater. Sci. Technol.* **27**, 607-614 (2011).
51. Wang, S. *et al.* Rational construction of hierarchically porous Fe–Co/N-doped carbon/rGO composites for broadband microwave absorption. *Nano Micro Lett.* **11**, 1-16 (2019).
52. Shi, G. *et al.* Enhanced microwave absorption properties of core double-shell type Fe@C@$BaTiO_3$ nanocapsules. *J. Alloys Compd.* **655**, 130-137 (2016).

53. Ding, L. *et al.* MIL-53 (Fe) derived MCC/rGO nanoparticles with excellent broadband microwave absorption properties. *Compos. Commun.* **21**, 100362 (2020).

54. Tong, Z. *et al.* Hierarchical Fe_3O_4/Fe@C@MoS_2 core-shell nanofibers for efficient microwave absorption. *Carbon* **179**, 646-654 (2021).

55. Ge, J. *et al.* 2D organic-α-Co $(OH)_2$ material and derived Co/C composites with bifunctions of anti-corrosion and microwave absorption. *Compos. Part B-Eng.* **224**, 109172 (2021).

56. Xiao, X. *et al.* Ultra-small Co/CNTs nanohybrid from metal organic framework with highly efficient microwave absorption. *Compos. Part B-Eng.* **152**, 316-323 (2018).

57. Li, D. *et al.* Microporous Co@C nanoparticles prepared by dealloying CoAl@C precursors: achieving strong wideband microwave absorption via controlling carbon shell thickness. *ACS Appl. Mater. Interfaces* **9**, 44704-44714 (2017).

58. Huang, W. *et al.* Hollow N-doped carbon polyhedra embedded Co and Mo_2C nanoparticles for high-efficiency and wideband microwave absorption. *Carbon* **167**, 19-30 (2020).

59. Ge, J. *et al.* Enhanced electromagnetic wave absorption of hybrid-architectures Co@SiO_xC. *J. Alloys Compd.* **831**, 154442 (2020).

60. Ma, J. *et al.* A facile fabrication and highly tunable microwave absorption of 3D flower-like Co_3O_4-rGO hybrid-architectures. *Chem. Eng. J.* **339**, 487-498 (2018).

61. Sha, L. *et al.* Chemical Ni–C bonding in Ni–carbon nanotube composite by a microwave welding method and its induced high-frequency radar frequency electromagnetic wave absorption. *ACS Appl. Mater. Interfaces* **9**, 40412-40419 (2017).

62. Zhou, C. *et al.* Rapid and direct growth of bipyramid TiO_2 from $Ti_3C_2T_x$ MXene to prepare Ni/TiO_2/C heterogeneous composites for high-performance microwave absorption. *Chem. Eng. J.* **383**, 123095 (2020).

63. Wang, L. *et al.* MOF-derived yolk-shell Ni@C@ZnO Schottky contact structure for enhanced microwave absorption. *Chem. Eng. J.* **383**, 123099 (2020).

64. Wang, L. *et al.* Synthesis and microwave absorption enhancement of Fe-doped NiO@SiO_2@graphene nanocomposites. *Mater. Sci. Eng. B-Adv.* **178**, 1403-1409 (2013).

65. Tong, Z. *et al.* Fabrication of flower-like surface Ni@Co_3O_4 nanowires anchored on RGO nanosheets for high-performance microwave absorption. *Appl. Surf. Sci.* **565**, 150483 (2021).

66. He, N. *et al.* Plasmon resonance strategy to enhance permittivity and microwave absorbing performance of Cu/C core-shell nanowires. *Chem. Eng. J.* **378**, 122160 (2019).

67. Qian, X. *et al.* Multi-path electron transfer in 1D double-shelled Sn@Mo_2C/C tubes with enhanced dielectric loss for boosting microwave absorption performance. *Small* **17**, 2100283 (2021).

68. Liu, J. *et al.* Self-assembled MoS_2/magnetic ferrite $CuFe_2O_4$ nanocomposite for high-efficiency microwave absorption. *Chem. Eng. J.* **429**, 132253 (2022).

69. Meng, X. *et al.* Carbon-coated defect-rich $MnFe_2O_4$/MnO heterojunction for high-performance microwave absorption. *Carbon* **194**, 207-219 (2022).

70. Zhao, H. Q. *et al.* Rational design of core-shell CoO@C nanotubes towards lightweight and high-efficiency microwave absorption. *Compos. Part B-Eng.* **196**, 108119 (2020).

71. Xu, R. X. *et al.* $CoFe_2O_4$/porous carbon nanosheet composites for broadband microwave absorption. *Chem. Eng. J.* **427**, 130796 (2022).

72. Yang, P. A. *et al.* Optimization of Fe@Ag core-shell nanowires with improved impedance matching and microwave absorption properties. *Chem. Eng. J.* **430**, 132878 (2022).

73. Chen, S. S. *et al.* Thermal conductivity of isotopically modified graphene. *Nat. Mater.* **11**, 203-207 (2012).

74. Malekpour, H. *et al.* Thermal conductivity of graphene laminate. *Nano Lett.* **9**, 5155-5161 (2014).

75. Zeng, W. *et al.* Defect-engineered reduced graphene oxide sheets with high electric conductivity and controlled thermal conductivity for soft and flexible wearable thermoelectric generators. *Nano Energy* **54**, 163-174 (2018).

76. Ma, W. G. *et al.* Chemically doped macroscopic graphene fibers with significantly enhanced thermoelectric properties. *Nano Res.* **11**, 741-750 (2018).

77. Chio, M. *et al.* Significantly enhanced thermoelectric performance of graphene through atomic-scale defect engineering via mobile hog-wire chemical vapor deposition systems. *ACS Appl. Mater. Interfaces* **13**, 24304 (2021).

78. Yao, J. A. *et al.* Enhanced thermoelectric properties of bilayer-like structural graphene quantum dots/single-walled carbon nanotubes hybrids. *ACS Appl. Mater. Interfaces* **12**, 39145 (2020).

79. Fujii, M. *et al.* Measuring the thermal conductivity of a single carbon nanotube. *Phys. Rev. Lett.* **95**, 065502 (2005).

80. Li, X. F. *et al.* Isotope-engineering the thermal conductivity of two-dimensional MoS_2. *ACS Nano* **13**, 2481-2489 (2019).

81. Gholivand H. *et al.* Effect of surface termination on the lattice thermal conductivity of monolayer $Ti_3C_2T_x$ MXenes. *J. Appl. Phys.* **126**, 065101 (2019).

82. Yuan K. P. *et al.* Effects of tensile strain and finite size on thermal conductivity in monolayer WSe_2. *Phys. Chem. Chem. Phys*. **21**, 468-477 (2019).

83. Santhosh, B. *et al.* Effect of pyrolysis temperature on the microstructure and thermal conductivity of polymer-derived monolithic and porous SiC ceramics. *J. Euro. Ceram. Soc.* **41**, 1151 (2021).

84. Zhao Y. J. *et al.* Honeycomb-like structured biological porous carbon encapsulating PED: A shape-stable phase change material with enhanced thermal conductivity for thermal energy storage. *Energy Buildings* **158**, 1049 (2018).

85. Kam, K. K. *et al.* Detailed photocurrent spectroscopy of the semiconducting group-VI transition metal chalcogenides. *J. Phys. Chem.* **86**, 463 (1982).

86. Peimyoo, N. *et al.* Thermal conductivity determination of suspended mono- and bilayer WS_2 by Raman spectroscopy. *Nano Res.* **8**, 1210 (2015).

87. Ujah, C. O. *et al.* Enhanced tribology, thermal and electrical properties of Al-CNT composite processed via spark plasma sintering for transmission conductor. *J. Mater. Sci.* **54**, 14064 (2019).

88. Mahapatra, P. *et al.* Seebeck coefficient of a single van der Waals junction in twisted bilayer graphene. *Nano Lett.* **17**, 6822-6827 (2017).

89. Lin Y. *et al.* Expression of interfacial Seebeck coefficient through grain boundary engineering with multi-layer graphene nanoplatelets. *Energy Environ. Sci.* **13**, 4114 (2020).

90. Xu, Y. *et al.* Thermal and thermoelectric properties of graphene. *Small* **10**, 2182 (2014).

91. Zheng, H. *et al.* Enhanced thermoelectric performance of graphene nanoribbons. *Appl. Phys. Lett.* **100**, 093104 (2012).

92. Chang, Y. *et al.* Synthesis process and thermoelectric properties of the layered crystal structure SnS_2. *J. Mater. Sci. Mater.-El.* **31**, 5425 (2020).

93. Chandra, S. *et al.* n-type ultrathin few-layer nanosheets of Bi-doped SnS_2: Synthesis and thermoelectric properties. *ACS Energy Lett.* **3**, 1153 (2018).

94. Karmakar, S. *et al.* First-principles prediction of enhanced thermoelectric properties of double transition metal MXenes: $Ti_{3-x}Mo_xC_2T_2$: (x=0.5, 1, 1.5, 2, 2.5, T=-OH/-O/-F). *Phys. Rev. Mater.* **4**, 124007 (2020).

95. Kong, S. *et al.* Realizing p-type MoS_2 with enhanced thermoelectric performance by embedding VMo_2S_4 nanoinclusions. *J. Phys. Chem. B* **122**, 713 (2018).

96. Gangwar, P. *et al.* Ultrahigh thermoelectric performance of 2H-MoS_2 nanosheets with incorporated conducting secondary phase. *Mater. Res. Express* **6**, 105062 (2019).

97. Cho, C. *et al.* stable n-type thermoelectric multilayer thin films with high power factor carbonaceous nanofillers. *Nano Energy* **28**, 426 (2016).

98. Lee, W. *et al.* Significant thermoelectric conversion efficiency enhancement of single layer graphene with substitutional silicon dopants. *Nano Energy* **87**, 106188 (2021).

99. Liu, X. *et al.* Flexible thermoelectric power generators fabricated using graphene/PEDOT:PSS nanocomposites films. *J. Mater. Sci. Mater.-El.* **30**, 20369 (2019).

100. Sadeghi, H. *et al.* Cross-plane enhanced thermoelectricity and phonon suppression in graphene/MoS_2 van der Waals heterostructures. *2D Mater*. **4**, 015012 (2016).

101. Wu, Q. Q. *et al.* Thermoelectricity in vertical graphene-C_{60}-graphene architectures. *Sci. Reps.* **7**, 11680 (2017).

102. Shin W. H. *et al.* Enhanced thermoelectric performance of reduced graphene oxide incorporated bismuth-antimony-telluride by lattice thermal conductivity reduction. *J. Alloys Compd.* **718**, 342 (2017).

103. Dong, D. P. *et al*. Assembling hollow carbon sphere-graphene polylithic aerogels for thermoelectric cells. *Nano Energy* **39**, 470 (2017).

104. Nunna, R. *et al.* Ultrahigh thermoelectric performance in Cu_2Se-based hybrid materials with highly dispersed molecular CNTs. *Energy Environ. Sci.* **10**, 1928 (2017).
105. Jagadish, P. *et al*. Recycled carbon fiber/Bi_2Te_3 and Bi_2S_3 hybrid composite doped with MWCNTs for thermoelectric applications. *Compos. Part B-Eng.* **175**, 107085 (2019).
106. Guan, X. *et al*. Significant enhancement in the Seebeck coefficient and power factor of *p*-type poly (3, 4-ethylenedioxythiophene):poly(styrenesulfonate) through the incorporation of *n*-type MXene. *ACS Appl. Mater. Interfaces* **12**, 13013 (2020).
107. Wang, X. C. *et al.* Achieving high thermoelectric performance of SnTe composites with 2D WSe_2. *J. Electron. Mater.* **51**, 486 (2022).
108. Reshak, A. *et al*. Thermoelectric properties of a single graphene sheet and its derivatives. *J. Mater. Chem. C* **2**, 2356 (2014).
109. Anno, Y. *et al*. Enhancement of graphene thermoelectric performance through defect engineering. *2D Mater*. **4**, 025019 (2017).
110. Ahmad, K. *et al*. Enhanced thermoelectric performance of Bi_2Te_3 based graphene nanocomposites. *Appl. Surf. Sci.* **2**, 474 (2019).
111. Feng, B *et al.* Enhanced thermoelectric properties of *p*-type $CoSb_3$/graphene nanocomposite. *J. Mater. Chem. A* **1**,13111 (2013).
112. Liang, B. B. *et.al* Fabrication and thermoelectric properties of graphene/Bi_2Te_3 composite materials. *J. Nanomater.* **6**, 6 (2013).
113. Chen, H.J. *et al.* Thermoelectric properties of $CuInTe_2$/graphene composites. *CrystEngComm* **15**, 6648 (2013).
114. Choo, S. *et al*. Cu_2Se-based thermoelectric cellular architectures for efficient and durable power generation. *Nat. Commun.* **12**, 3550 (2021).
115. Luo, Y.B. et al. Cubic $AgMnSbTe_3$ semiconductor with a high thermoelectric performance. *J. Am. Chem. Soc.* 143, 13990 (2021).
116. Luo, Z.Z. *et al*. Extraordinary role of Zn in enhancing thermoelectric performance of Ga-doped n-type PbTe. *Energy Environ. Sci.* **15**, 368 (2022).
117. Ma, Z. *et al.* High entropy semiconductor $AgMnGeSbTe_4$ with desirable thermoelectric performance. *Adv. Funct. Mater.* **21**, 2103197 (2013).
118. Hu, L. *et al.* High thermoelectric performance enabled by convergence of nested conduction bands in $Pb_7Bi_4Se_{13}$ with low thermal conductivity. *Nat. Commun.* **12**, 4793 (2021).
119. Zhang, Q. *et al*. High-performance thermoelectric material and module driven by medium-entropy engineering in SnTe. *Adv. Funct. Mater.* **32**, 220548 (2022).
120. Hu, H.H. *et al*. Thermoelectric $Cu_{12}Sb_4S_{13}$-based synthetic minerals with a sublimation-derived porous network. *Adv. Mater*. **33**, 2103633 (2021).
121. Hu, L. *et al*. High thermoelectric performance through crystal symmetry enhancement in triply doped diamondoid compound Cu_2SnSe_3. *Adv. Energy Mater.* **11**, 2100661 (2021).
122. Luo, Y. B. *et al*. Thermoelectric performance of 2D $Bi_2Si_2Te_6$ Semiconductor. *J. Am.*

Chem. Soc. **144**, 1445 (2022).

123. Wang, L.Y. *et al*. Discovery of a slater-pauling semiconductor $ZrRu_{1.5}Sb$ with promising thermoelectric properties. *Adv. Funct. Mater*. **32**, 2200438 (2022).

124. Sheng, M. *et al.* Significant enhanced thermoelectric performance in SWCNT films via carrier tuning for high power generation. *Carbon* **158**, 802-807 (2020).

125. Wu, Y. *et al.* Triboelectric-thermoelectric hybrid nanogenerator for harvesting energy from ambient environments. *Adv. Mater. Technol.* **3**, 1800166 (2018).

126. Ding, Y. F. *et al.* High performance *n*-type Ag_2Se film on nylon membrane for flexible thermoelectric power generator. *Nat. Commun.* **10**, 841 (2019).

127. Lu, Y. *et al.* Good performance and flexible PEDOT:PSS/Cu_2Se nanowire thermoelectric composite films. *ACS Appl. Mater. Interfaces* **11**, 12819-12829 (2019).

128. Bae E. *et al.* Enhancement of thermoelectric properties of PEDOT:PSS and Tellurium-PEDOT:PSS hybrid composites by simple chemical treatment. *Sci. Rep.* **6**, 18805 (2016).

129. Wang, L. M. *et al.* Engineering carrier scattering at the interfaces in polyaniline based nanocomposites for high thermoelectric performances. *Mater. Chem. Front.* **1**, 741-748 (2017).

130. Tian, R. *et al.* A solution-processed TiS_2/organic hybrid superlattice film towards flexible thermoelectric devices. *J. Mater. Chem. A* **5**, 564-570 (2017).

131. Lu, Y. *et al.* Preparation and characterization of Te/poly (3, 4-ethylenedioxythiophene): poly(styrenesulfonate)/Cu_7Te_4 ternary composite films for flexible thermoelectric power generator. *ACS Appl. Mater. Interfaces* **10**, 42310 (2018).

132. Meng, Q. F. *et al.* High performance and flexible polyvinylpyrrolidone/Ag/Ag_2Te ternary composite film for thermoelectric power generator. *ACS Appl. Mater. Interfaces* **11**, 33254 (2019).

133. Wang, Z. X. *et al.* High performance Ag_2Se/Ag/PEDOT composite films for wearable thermoelectric power generators. *Mater. Today Phys.* **21**, 100553 (2021).

134. Wang, L. M. *et al.* Textile-based thermoelectric generators and their applications. *Energy Environ. Mater.* **3**, 67-79 (2019).

135. Romanenko, A. *et al.* Electron transport properties of thermoelectric based on layered substituted transition metal dichalcogenides. *J. Structure Chem.* **58**, 893 (2017).

136. Koyano, M. *et al.* Single-crystal growth of Bi-Sb-Te thermoelectric materials by halide chemical vapor transport technique. *J. Electron. Mater*. **41**, 1317 (2012).

137. Yu, J. J. *et al.* Half-heusler thermoelectric module with high conversion efficiency and high power density. *Adv. Energy Mater*. **10**, 2000888 (2020).

138. Mallick, M. M. *et al.* High-performance Ag-Se-based n-type printed thermoelectric materials for high power density folded generators. *ACS Appl. Mater. Interfaces* **12**, 19655 (2020).

139. Yu, C. X. *et al.* Strain engineering on the thermal conductivity and heat flux of thermoelectric Bi_2Te_3 nanofilm. *Nano Energy* **17**, 104 (2014).

140. Tan, G. J. *et al.* Thermoelectric performance optimization in *p*-type $Ce_yFe_3CoSb_{12}$ skutterudites. *J. Electron Mater.* **43**, 1712 (2014).

141. Saberi, Y. *et al.* A comprehensive review on the effects of doping process on the thermoelectric properties of Bi_2Te_3 based alloys. *J. Alloys Compd.* **904**, 163918 (2022).

142. Gurbanov, G. R. *et al.* Physicochemical interactions in the $GeSb_2Te_4$-$PbSb_2Te_4$ system. *Semiconductor* **54**, 1304 (2020).

143. Nielsen, M. D. *et al.* Off-stoichiometric silver antimony telluride: An experimental study of transport properties with intrinsic and extrinsic doping. *AIP Advance* **5**, 053602 (2015).

144. Chu J. *et al*. Electrode interface optimization advances conversion efficiency and stability of thermoelectric device. *Nat. Commun.* **11**, 2723 (2020).

145. Zong, P. A. *et al.* Skutterudite with graphene-modified grain-boundary complexion enhances ZT enabling high-efficiency thermoelectric device. *Energy Environ. Sci.* **10**, 183 (2017).

146. Zhu Q. *et al.* Realizing high conversion efficiency of Mg_3Sb_2-based thermoelectric materials. *J. Power Sources.* **414**, 393 (2019).

147. Liu Z.H. *et al.* Demonstration of ultrahigh thermoelectric efficiency of ~7.3% in Mg_3Sb_2/MgAgSb module for low-temperature energy harvesting. *Joule* **5**, 1196 (2021).

148. Kraemer, D. et al. High thermoelectric conversion efficiency of MgAgSb-based material with hot-pressed contacts. *Energy Environ. Sci.* **5**, 1299 (2015).

149. Qiu P. F. *et al.* High-efficiency and stable thermoelectric module based on liquid-like materials. *Joule* **3**, 1538 (2019).

150. Zhu, H.T. *et al.* Discovery of ZrCoBi based half Heuslers with high thermoelectric conversion efficiency. *Nat. Commun.* **9**, 2497 (2018).

151. Zhu, H.T. *et al.* Discovery of TaFeSb-based half-Heuslers with high thermoelectric performance. *Nat. Commun.* 10, 270 (2019).

152. Pan, Y. *et al.* Melt-centrifuged $(Bi,Sb)_2Te_3$:Engineering microstructure toward high thermoelectric efficiency. *Adv. Mater.* **30**, 1802016 (2018).

153. Salvador, J. R. et al. Conversion efficiency of skutterudite-based thermoelectric modules. *Phys. Chem. Chem. Phys.* **16**, 12510 (2014).

154. Hsu, C.T. et al. An effective Seebeck coefficient obtained by experimental results of a thermoelectric generator module. *Appl. Energy* **88**, 5173 (2011).
